# Microbiota Transplantation Among Patients Receiving Long-Term Care: The Sentinel REACT Nonrandomized Clinical Trial

**DOI:** 10.1001/jamanetworkopen.2025.22740

**Published:** 2025-07-24

**Authors:** Michael H. Woodworth, Ahmed Babiker, Radhika Prakash-Asrani, C. Christina Mehta, Danielle Barrios Steed, Amanda Ashley, Dylan Koundakjian, Adi Acharya, Lori Grooms, Chris W. Bower, Deepti R. Suchindran, Twinkle Trehan, Alison Laufer Halpin, Maroya Spalding Walters, Sujan C. Reddy, Matthew H. Samore, Mary-Claire Roghmann, Mary K. Hayden, Julia Van Riel, Eileen M. Burd, Sarah Lohsen, Sarah W. Satola, Scott K. Fridkin

**Affiliations:** 1 Division of Infectious Diseases, Department of Medicine, Emory University School of Medicine, Atlanta, Georgia; 2 Division of Hospital Medicine, Department of Medicine, Emory University School of Medicine, Atlanta, Georgia; 3 Emory Long Term Acute Care, Decatur, Georgia; 4 Division of Healthcare Quality Promotion, Centers for Disease Control and Prevention, Atlanta, Georgia; 5 Department of Pathology, Emory University School of Medicine, Atlanta, Georgia; 6 Division of Epidemiology, Department of Internal Medicine, University of Utah, Salt Lake City; 7 Department of Epidemiology and Public Health, University of Maryland School of Medicine, Baltimore; 8 Department of Internal Medicine, Rush University Medical Center, Chicago, Illinois

## Abstract

**Importance:**

Intestinal multidrug-resistant organism (MDRO) colonization is highly prevalent in long-term acute care hospital (LTACH) patients and is associated with MDRO infection and transmission. However, there are no therapies approved by the US Food and Drug Administration to reduce intestinal MDRO colonization.

**Objective:**

To determine the safety and acceptability of fecal microbiota transplantation (FMT) in LTACH patients.

**Design, Setting, and Participants:**

This single-center, open-label nonrandomized clinical trial was conducted from April to December 2023 at an LTACH in the Southeastern US with median 50-patient census and 28-day length of stay. Patients with MDRO colonization were identified by perirectal prevalence sampling. Patients colonized with at least 1 target MDRO were approached for informed consent for FMT. FMT recipients were compared with untreated controls with MDRO colonization. Data were analyzed from August 2024 to May 2025.

**Intervention:**

Healthy donor fecal microbiota (50-100 g stool and 250 mL normal saline with 9% glycerol) instilled via gastrostomy tube or enema without antibiotic or bowel preparation conditioning.

**Main Outcomes and Measures:**

The primary outcome was frequency and severity of adverse events. Solicited adverse events were recorded for 7 days. Unsolicited adverse events were recorded for 6 months. Four weekly perirectal MDRO cultures were performed after FMT.

**Results:**

A total of 42 patients, including 10 (mean [SD] age, 63.8 (14.5) years; 7 [70%] female) who received FMT and 32 contemporaneous controls (mean [SD] age, 64.0 [13.7] years; 13 [41%] female) were assessed. In 2 prevalence surveys, 23 of 32 (72%) and 26 of 34 (77%) perirectal cultures grew at least 1 MDRO. Among the FMT group, 5 patients received FMT via gastrostomy alone, 4 via enema alone, and 1 with both routes more than 30 days apart. No serious adverse events were attributed to FMT, and post-FMT solicited adverse events were mild. At final visit, all perirectal cultures from FMT recipients grew at least 1 MDRO. Post hoc analyses found numerically fewer FMT recipients had positive blood culture results (0 individuals vs 6 individuals [19%]; *P* = .31), pathogen intestinal dominance (2 of 8 individuals [25%] vs 4 of 8 individuals [50%]; *P* = .61), and 7 fewer days of antibiotic therapy per 1000 patient days (median [IQR], 12.6 [0-25.2] days vs 19.7 [6.5-36.1] days; *P* = .38) compared with controls in the 6 months after prevalence survey, although these differences were not statistically significant. Accounting for higher baseline FMT recipient antibiotic use, difference-in-differences analysis estimated 26 (95% CI, −64 to 12) fewer days of antibiotic therapy per 1000 patient-days after FMT, although this difference was also not statistically significant.

**Conclusions and Relevance:**

In this nonrandomized pilot clinical trial, FMT was acceptable for LTACH patients without related serious adverse events. Although not powered to test these outcomes, this study found potential reductions in bacteremia, intestinal pathogen domination, and antibiotic use associated with FMT, suggesting FMT should be evaluated in larger, randomized trials.

**Trial Registration:**

ClinicalTrials.gov Identifier: NCT05780801

## Key Points

**Question:**

Is fecal microbiota transplantation (FMT) acceptable and safe for long-term acute care hospital patients with intestinal multidrug-resistant organism (MDRO) colonization?

**Findings:**

In this nonrandomized pilot clinical trial of 42 patients, including 10 who received FMT and 32 contemporaneous controls, no FMT recipients had severe adverse events related to the procedure or material. No FMT recipients had positive blood cultures in the 6 months after treatment compared with 19% of controls, although the difference did not reach statistical significance.

**Meaning:**

This nonrandomized clinical trial found that FMT administered via gastrostomy or enema instillation was well tolerated in long-term acute care hospital patients; these findings suggest that FMT warrants further study for infection prevention in larger randomized trials.

## Teaser

This nonrandomized clinical trial examines the safety and acceptability of fecal microbiota transplantation in long-term acute care hospital patients with perirectal culture results positive for multidrug-resistant organisms.

## Introduction

Antimicrobial resistance remains a global threat.^1^ Intestinal colonization with multidrug-resistant organisms (MDROs) has been associated with increased risk of infection and transmission, particularly among patients recovering from critical illness in long-term care facilities, such as long-term acute care hospitals (LTACHs).^2^ Despite these challenges, there are no therapies to reduce intestinal MDRO colonization that have been approved by the US Food and Drug Administration (FDA). This gap was highlighted as an urgent priority in a joint US Centers for Disease Control and Prevention and FDA workshop held in August 2022,^3^ which underscored the critical need to develop treatments to reduce pathogen colonization with an emphasis on the central role of the microbiome in MDRO colonization.

Prior studies have reported an association of increased intestinal pathogen relative abundance with risk of subsequent bloodstream and urinary tract infections, suggesting that reducing intestinal pathogen relative abundance could reduce risk of infection.^4,5,6^ A 2021 study^7^ also found that microbiome interventions, such as fecal microbiota transplantation (FMT), are associated with reduced frequency of infection, days of antibiotic use, and length of stay, even when MDROs are still detectable by culture. In addition to reduced intestinal MDRO colonization, microbiome interventions have also been associated with increased all-cause survival at 90 days in multiple studies,^8,9,10,11,12^ but to our knowledge, these interventions have not been evaluated in patients hospitalized in LTACHs, facilities that care for patient populations with complex comorbidities and a high prevalence of MDRO colonization. A pilot study conducted in nursing home residents suggested that autologous FMT is not feasible, largely due to difficulty banking baseline stool samples.^13^ Allogeneic FMT from screened donors has been feasible for other indications and populations, but to our knowledge, no microbiota therapeutic studies have been conducted in LTACHs. Although FMT has been administered to more than 65 000 patients, to our knowledge, there are no safety data available for LTACH patients with MDRO colonization.^14^ This population also relies heavily on legally authorized representatives to make their health care decisions, and to our knowledge, there are no data to assess acceptability of FMT to this population or their surrogate decision-makers. The Sentinel Response to Emerging Antimicrobial Resistance with Containment Microbiota Restoration Therapy (REACT) trial was conducted to evaluate the safety and acceptability of FMT in patients admitted to an LTACH to inform planning for a larger phase 2 study.

## Methods

### Study Design

The Sentinel REACT trial was an open-label, nonrandomized pilot clinical trial with 6 months of follow-up conducted at a single academic-affiliated LTACH in Atlanta, Georgia, from April to December 2023. The trial protocol was reviewed as part of an FDA research investigational new drug application and approved by the Emory University institutional review board under 45 CFR part 46.101(c) and 21 CFR part 56. Potentially eligible participants (or their legally authorized representative) with MDRO-positive results willing to undergo treatment signed an electronic informed consent form to participate in Sentinel REACT. A data safety monitoring board reviewed enrollment, safety, efficacy, and death data after the first participant group was treated and every 6 months until safety follow-up was complete. This study is reported according to the Transparent Reporting of Evaluations With Nonrandomized Designs (TREND) reporting guideline for nonrandomized trials.^15^ The trial protocol and statistical analysis plan are included in Supplement 1.

To identify participants with MDRO colonization and estimate LTACH MDRO prevalence, all admitted patients underwent inguinal and perirectal cultures with verbal consent on a single day under a separate institutional review board–approved Antimicrobial Resistance Point Prevalence Sampling Protocol (APPS). MDROs of interest included extended-spectrum β-lactamase–producing *Enterobacterales*, carbapenem-resistant *Enterobacterales*, multidrug-resistant *Pseudomonas*, vancomycin-resistant *Enterococcus* species, and toxigenic *Clostridioides difficile* (eMethods in Supplement 2). Admitted patients with MDRO-positive perirectal culture findings were reapproached for informed consent discussions for FMT in the Sentinel REACT trial. Eligible participants from each prevalence survey were treated on a single day following that survey. The aim was to administer FMT to at least 10 participants.

### Study Outcomes

The primary outcome was frequency and severity of adverse events (AEs), classified by Common Terminology Criteria for Adverse Events version 5.0. The secondary outcome was proportion of participants with positive MDRO perirectal or stool culture results at week 2 and week 4 after FMT. Exploratory outcomes included frequency of infection in the 6 months prior to screening compared with 6 months after screening and metagenomic analyses of pathogen relative abundance trends. Post hoc tests of positive clinical microbiology results, days of antibiotic therapy, α diversity, and proportion of patients with intestinal domination defined by genus-level pathogen relative abundance of at least 30% were performed comparing FMT recipients to contemporaneous controls who did not receive FMT. Infection analyses were not restricted to MDRO infections because potential microbiota-mediated reductions in pathogen burden and improved gut barrier integrity would be anticipated to reduce infection regardless of antibiotic susceptibility profile.

### Recruitment and Eligibility Criteria

All admitted patients, or their legally authorized representatives, were contacted in APPS to obtain verbal consent for perirectal and inguinal sampling for MDRO culture. Potentially eligible patients with MDRO-positive results were excluded from participation in Sentinel REACT if they had uncontrolled intercurrent illness, were prescribed systemic antibiotics that would not be complete prior to FMT administration, were unable to discontinue proton-pump inhibitor therapy, were pregnant or immunocompromised, had a history of food allergy leading to anaphylaxis or hospitalization, or had a life expectancy of 24 weeks or less. Participants with history of grade III or IV hemorrhoids or uncontrolled hemorrhoid pain or bleeding were excluded from enema FMT administration. If participants were discharged prior to completion of scheduled study visits, they were contacted by study staff to ascertain interim AEs.

### Intervention

The FMT group received healthy donor fecal microbiota (50-100 g of stool suspended in 250 mL normal saline with 9% glycerol by final volume). Fecal microbiota was instilled via gastrostomy tube or enema without antibiotic or bowel preparation conditioning.

### Controls

FMT recipients were compared with contemporaneous controls with MDRO-positive results who were sampled in APPS on the same days but did not receive FMT. For post hoc analyses of outcomes in clinical cultures, antibiotic use, and disposition, FMT recipients were compared with all untreated participants with MDRO-positive results in at least 1 APPS prevalence survey. For post hoc metagenomic analyses, FMT recipients were compared with all untreated participants with MDRO-positive results sampled in 2 APPS prevalence surveys.

### Data Collection and Outcome Assignment

Participant demographic characteristics (eg, sex, race, age) were abstracted from the medical record. Race data, categorized as Black, White, or other or unknown (including Korean, Other Pacific Islander, decline to answer), were abstracted as a surrogate indicator of sociodemographic exposures, not biological effects. Solicited AEs (ie, abdominal pain, fever, diarrhea, constipation, flatulence, bloating, vomiting) were assessed for FMT recipients daily at baseline (day 0 prior to treatment), immediately after treatment, and days 1 to 7 by participant interview or discussion with the participant’s nurse. Unsolicited AEs were assessed for FMT recipients weekly from day 7 to day 28. Severe AEs and AEs of special interest were assessed monthly until 6 months after the last visit (day 208). Noninfection AEs were not systematically abstracted for contemporaneous controls sampled in APPS. AEs were classified as potentially related if the event occurred after treatment, probably related if there was evidence to suggest a causal relationship and influence of other factors was unlikely, and definitely related if there was clear evidence to suggest a causal relationship and other possible contributing factors could be ruled out. Exploratory clinical outcomes of antibiotic days of therapy, clinical microbiology results, and final disposition at last encounter or 6 months from prevalence sampling in APPS were assessed by duplicate medical record review by trained clinical reviewers (M.H.W., and D.K., and T.T.). All positive bacterial clinical microbiology results and antibiotic doses were abstracted for the 6 months preceding and following the date of perirectal and inguinal prevalence sampling under APPS.

### Statistical Analysis

Patient characteristics, AEs, culture results, and measures of health care utilization were described in tabular summaries. Given the cohort size, differences in central tendency were tested by the Wilcoxon rank-sum test, and proportions were tested by Fisher exact test. The magnitude and direction of associations were described and compared to inform effect size estimates for future studies, as the sample size of cohort limited power for formal hypothesis tests. *P* values were 2-sided, and statistical significance was set at *P* ≤ .05. Data were analyzed using RStudio version 2024.04.2+764 (R Project for Statistical Computing) from August 2024 to May 2025.

## Results

### MDRO Prevalence and Study Participants Recruited

**Figure 1.**
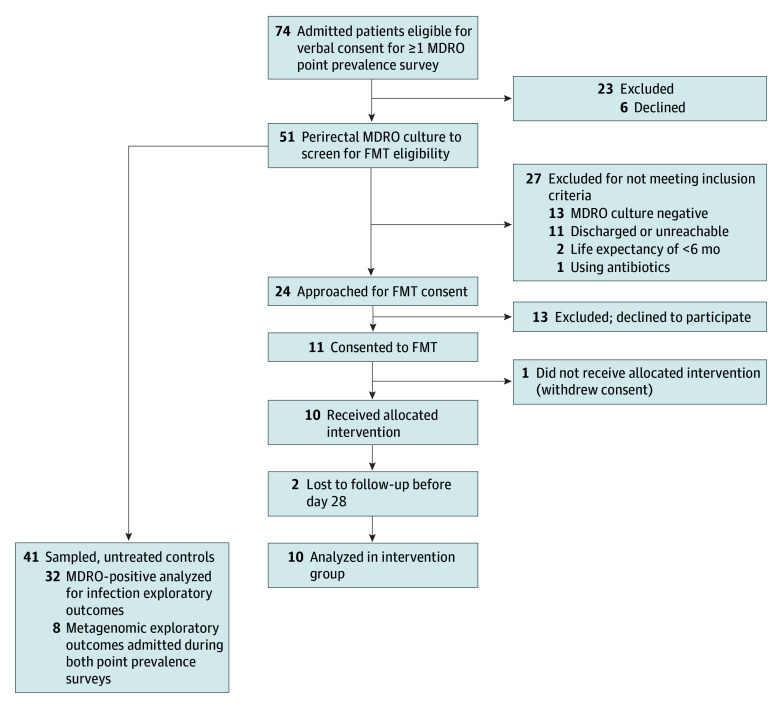
Flowchart of Participant Recruitment, Treatment Allocation, and Analysis Controls were admitted to the same facility at the same time and underwent peri-rectal cultures but did not meet inclusion criteria or declined to participate and did not receive fecal microbiota transplant (FMT). Metagenomic exploratory outcome group was a subset of the controls with culture results positive for multidrug-resistant organisms (MDRO) analyzed in the infection exploratory outcome group.

Perirectal and inguinal swabs were collected from 32 of 43 (74%) and 34 of 42 (81%) of admitted patients, respectively, representing 51 unique participants (25 [49%] female; mean [SD] age, 63.0 [13.6] years). These 51 participants were sampled in 2 prevalence surveys conducted 35 days apart in 2023 at the Emory Long Term Acute Care Hospital. The flow of participants from screening through the end of study is presented in Figure 1. A total of 23 of 32 (72%) and 26 of 34 (77%) perirectal swabs grew at least 1 MDRO in the 2 prevalence surveys (eFigure 1 in Supplement 2). A total of 27 of 32 participants in prevalence survey 1 and 32 of 34 participants in prevalence survey 2 were sampled after obtaining verbal consent from a legally authorized representative. Ten participants with MRDO consented to FMT, with 8 of them (80%) participating with consent obtained from a legally authorized representative. The final sample included 42 patients, with 10 (mean [SD] age, 63.8 (14.5) years; 7 [70%] female) receiving FMT and 32 contemporaneous controls (mean [SD] age, 64.0 [13.7] years; 13 [41%] female).

There were 6 participants with MDRO-positive cultures treated with FMT via gastrostomy tube instillation after the first prevalence survey and 5 participants treated via enema after the second prevalence survey. This represented treatment of 11% to 14% of the patients hospitalized at the facility on a single day. One participant (SNT-01) vomited after instillation of the FMT via gastrostomy tube. SNT-01 was still hospitalized on the date of the second prevalence survey, provided updated informed consent, was rescreened with MDRO-positive results, and treated with a second FMT via enema (as SNT-13). The 6 months of follow-up for AEs was completed in December 2023. Baseline demographic and clinical characteristics for these 10 unique FMT recipients and the 32 controls who were not treated with FMT are shown in Table 1. Two participants were lost to follow-up after day 28 before completing 6 months of AE monitoring (SNT-03 and SNT-06). The size of this cohort limited power to meaningfully compare differences in efficacy by gastrostomy tube instillation and enema administration routes in this population.

**Table 1.** Demographic and Baseline Clinical Characteristics of Fecal Microbiota Transplant (FMT) Recipients Compared With Contemporaneous Controls Who Did Not Receive FMT

Characteristic	Individuals, No. (%)^a^	
	FMT Recipients (n = 10)	Controls (n = 32)
Age, mean (SD), y	63.8 (14.5)	64.0 (13.7)
Sex		
Male	3 (30)	19 (59)
Female	7 (70)	13 (41)
Race		
Black	5 (50)	18 (56)
White	3 (30)	5 (16)
Other or unknown^b^	2 (20)	9 (28)
Medical history^c^		
Diabetes	4 (40)	11 (34)
Hemodialysis	2 (20)	5 (16)
Length of admission, median (IQR), d	26 (19-52)	24 (12-46)
Peri-rectal culture MDRO Positivity^d^		
CRE	0	9 (28)
* C difficile*	3 (30)	6 (19)
ESBL	4 (40)	14 (44)
MDRP	3 (30)	3 (9)
VRE	5 (50)	14 (49)

Abbreviations: *C difficile*, toxigenic *Clostridioides difficile*; CRE, carbapenem-resistant *Enterobacteriaceae*; ESBL, extended-spectrum β-lactamase–producing *Enterobacterales*; MDRO, multidrug-resistant organism; MDRP, multidrug-resistant *Pseudomonas* species*; *VRE, vancomycin-resistant *Enterococcus* species.

^a^ All participants had positive results for multidrug resistant organisms in perirectal swab sampling.

^b^ Race categories listed as other include Korean, Other Pacific Islander, and decline to answer.

^c^ No patients had a history of inflammatory bowel disease or active treatment of malignanct neoplasm with chemotherapy.

^d^ MDRO-positive culture results are not mutually exclusive.

### Safety

No severe AEs were classified as probably or definitely related to FMT treatment (eTable 1 in Supplement 2). Solicited adverse events after FMT were generally mild (eTable 2 in Supplement 2). Several participants developed AEs that were not classified as probably or definitely related to FMT treatment, which were largely driven by the medical complexity of this population, with most of these AEs occurring in 3 participants. The most notable nonsevere AE was vomiting after upper GI administration (SNT-01). Two participants (SNT-02 and SNT-08) died after receiving FMT. SNT-02 was deemed unlikely to be weaned from mechanical ventilatory support and died prior to collection of the day 28 culture without any noted signs or symptoms of new infection or fever. SNT-08 died before specimens could be collected on days 21 and 28 after developing progressive respiratory failure, which was similar to a prior event related to medical history of valvular heart failure prior to FMT. These deaths were not classified as probably or definitely related to FMT administration. Review of these deaths by the data safety monitoring board agreed with this assessment.

### MDRO Colonization

**Figure 2.**
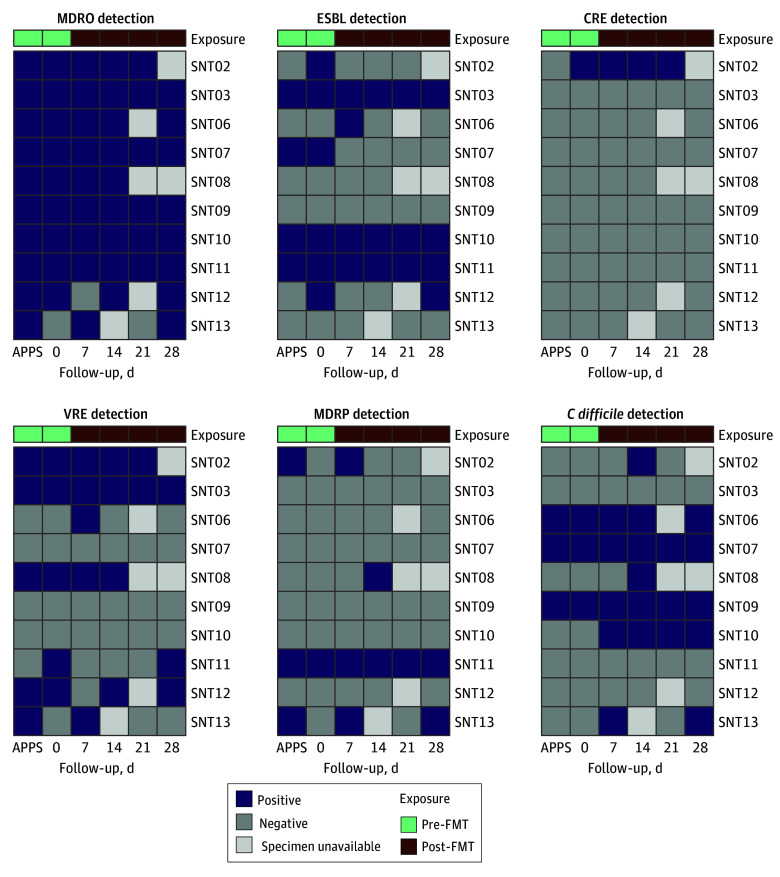
Perirectal Multidrug-Resistant Organism (MDRO) Culture Results by Visit Among 10 Fecal Microbiota Transplant (FMT) Recipients APPS indicates Antimicrobial Resistance Point Prevalence Sampling Protocol; CRE, carbapenem-resistant *Enterobacterales*; ESBL, extended-spectrum β-lactamase; FMT, fecal microbiota transplant; MDRP, multidrug-resistant *Pseudomonas*; VRE, vancomycin-resistant *Enterococcus*. MDRO detection is a summary of composite detection of all MDRO categories.

Three of 10 participants (30%) with at least 1 extended-spectrum β-lactamase–producing *Enterobacterales*–positive perirectal culture and 2 of 10 participants (20%) with at least 1 vancomycin-resistant *Enterococcus–*positive perirectal culture did not grow isolates in these categories at their last visit. Several participants (6 of 10 participants [60%]) had a new MDRO category detected by perirectal culture during the study that was not detected by the APPS prevalence sample. All perirectal culture results from FMT recipients remained positive with at least 1 MDRO at days 14 and 28 (Figure 2).

### Microbiome Dynamics Detected by Perirectal Swab Shotgun Metagenomic Sequencing

**Figure 3.**
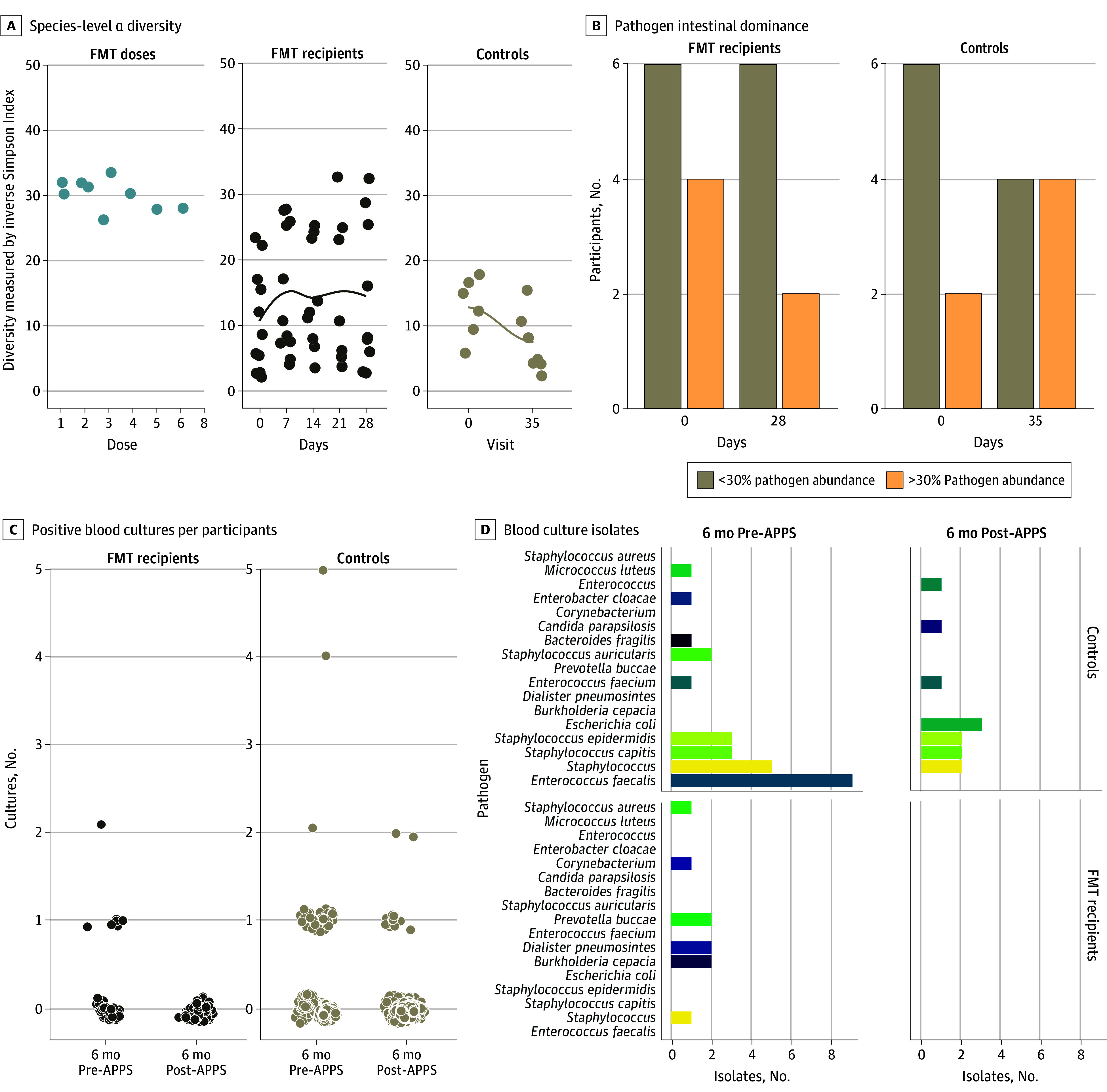
Blood Culture and Microbiome Trends After Fecal Microbiota Transplant (FMT) FMT recipients (n = 10) had a more stable α diversity (A) and decrease in number of participants with pathogen intestinal microbiome dominance (note multiple FMT doses are made from individual stool specimens) (B) compared with untreated contemporaneous controls (n = 32), although these differences were not statistically significant. FMT recipients also had fewer positive blood culture results in the 6 months after prevalence survey (C and D) compared with controls with MDRO-positive blood culture results by perirectal prevalence survey but did not receive FMT, although these differences were not statistically significant. A, The horizontal axis for FMT doses indicates aliquots from 2 donors that represent the 11 administered doses. Metagenomic analysis (A and B) controls were all individuals who were admitted on both prevalence surveys 35 days apart under the Antimicrobial Resistance Point Prevalence Sampling protocol (n = 8).

As shown in Figure 3A, species-level α diversity measured by the Inverse Simpson Index was highest in the FMT doses, with a mean (SD) of 30.1 (2.4). We observed increased α diversity in FMT recipients from a baseline mean (SD) of 10.6 (8.2) on day 0 to 16.0 (11.4) on day 28. Mean (SD) α diversity decreased in 8 untreated controls with MDRO-positive results sampled on both days from 11.8 (7.4) at the time of the first prevalence sample to 6.4 (4.6) at the second prevalence sample (Wilcoxon rank-sum *P* = .11). The percentage of living FMT recipients with intestinal domination decreased at follow up from 40% (4 of 10 participants) to 25% (2 of 8 participants), while the percentage of untreated controls with intestinal domination increased at follow up from 25% (2 of 8 participants) to 50% (4 of 8 participants) (Figure 3B).^5^ Of the 2 participants who died, SNT-08 did not have baseline intestinal domination and SNT-02 had baseline intestinal domination with *Enterococcus* that was not subsequently noted in day 7, 14, and 21 samples collected after FMT and before death.

### Clinical Outcomes

In the 6 months prior to first prevalence sample, FMT recipients and controls had a similar number of positive clinical microbiology culture findings and median days of antibiotic therapy (Table 2 and Figure 3C and D; eFigure 2 and eFigure 3 in Supplement 2). No FMT recipients had any positive blood culture results in the 6 months after sampling, whereas 6 untreated controls (19%) had positive blood culture results (*P* = .31). FMT recipients also had numerically fewer positive specimen types other than blood, urine, or respiratory samples compared with untreated contemporaneous controls, although this difference was not statistically significant (Table 2; eFigure 4 in Supplement 2). Post hoc analyses found FMT recipients had numerically fewer days of antibiotic therapy per 1000 patient days (median [IQR], 12.6 [0-25.2] days vs 19.7 [6.5-36.1] days; *P* = .38) compared with controls in the 6 months after prevalence survey, although this difference was not statistically significant. A higher proportion of FMT recipients (5 of 10 participants [50%]) compared with controls (12 of 32 participants [38%]) were residing at home at final follow-up encounter, with median (IQR) of 180 (72-180) days of follow-up for the treated group and 180 (143-180) days for the untreated group. Difference-in-differences analysis of days of antibiotic therapy accounting for baseline group characteristics found FMT recipients compared with untreated controls had a mean of 26 (95% CI, −64 to 12) fewer days of antibiotic therapy in the 6 months post-APPS sampling compared with the 6 months pre-APPS sampling, although this difference was not statistically significant (eTable 3 and eTable 4 in Supplement 1).

**Table 2.** Clinical Characteristics of FMT Recipients Compared With Contemporaneous Controls Who Did Not Receive FMT

Characteristic	Individuals, No. (%)^a^		*P* value^b^
	FMT (n = 10)	MDRO-positive controls (n = 32)	
Positive clinical microbiology results in 6 mo before sampling			
Blood	4 (40)	12 (38)	>.99
Urine	3 (30)	12 (38)	>.99
Respiratory	7 (70)	20 (63)	>.99
Other	3 (30)	10 (31)	>.99
Participants with microbiology results in 6 mo after sampling			
Blood	0	6 (19)	.31
Urine	3 (30)	8 (25)	>.99
Respiratory	3 (30)	13 (41)	.72
Other	0	8 (25)	.17
Antibiotic therapy rate, median (IQR), d per 1000 patient-days			
6 mo before sampling	35.3 (10.6-69.6)	24.4 (13.8-38.1)	.66
6 mo after sampling	12.6 (0-25.2)	19.7 (6.5-36.1)	.38
Final disposition at 6 mo after sampling			
Acute care hospital	0	2 (6)	.72
Home	5 (50)	12 (38)	
Rehabilitation or skilled nursing facility	3 (30)	8 (25)	
Long-term acute care hospital	0	5 (16)	
Deceased or hospice	2 (20)	5 (16)	
Postintervention follow up time, median (IQR), d	180 (72-180)	180 (143-180)	.83

^a^ All participants had positive results for multidrug resistant organisms in perirectal swab sampling.

^b^ Wilcoxon rank-sum test for continuous variables and Fisher exact test for categorical variables.

## Discussion

In this nonrandomized pilot clinical trial, a single FMT in patients with MDRO colonization without antibiotic or bowel-preparation conditioning was feasible without related severe AEs in LTACH patients. While the observed MDRO decolonization rates after FMT were lower in this feasibility and safety study compared with other reports,^8,10,16^ FMT-treated participants had numerically fewer episodes of bacteremia, decreased pathogen intestinal dominance, and fewer days of antibiotic therapy compared with controls, although these findings did not reach statistical significance.

Prior clinical studies of FMT for patients with MDRO-positive culture results have reported variable decolonization effect sizes.^8,17,18,19^ This variability may be related to differences in number of doses administered, routes of administration (eg, gastric instillation, enema, capsules), conditioning regimens (eg, polyethylene glycol, vancomycin), patient populations studied, and definitions of response.^20^ The FEDEX study, which did not use antibiotic conditioning prior to duodenal FMT instillation, also observed lower frequency of MDRO decolonization.^21^ A study of 8 *Clostridial* strains to prevent *C difficile* recurrence found increased engraftment with conditioning with vancomycin and with extended dosing over several days.^22^ When the phase 2 clinical trial of fecal microbiota spores live-brpk (SER-109) for prevention of recurrent *C difficile* did not meet prespecified end points, an increased dose in the phase 3 trial successfully met end points and supported FDA approval.^23,24^ Participants in Sentinel REACT who were treated with a single FMT without antibiotic or bowel preparation conditioning had higher mean α diversity at last visit compared with contemporaneous controls, although the difference was not statistically significant. However, the conversion to MDRO negative perirectal swab culture results was less frequent in this study than prior reports. The feasibility and modest efficacy of a single FMT suggest that subsequent studies should explore whether increased doses, more frequent dosing, or conditioning regimens with antibiotics or laxatives (which may be less feasible in LTACH patients) increase efficacy of decolonization.

Observational studies suggest an increased risk of blood stream infection and urinary tract infection for patients above pathogen intestinal microbiome relative abundance thresholds.^4,5,6,25,26^ Other studies have reported that FMT is associated with clinically meaningful benefits of reduced mortality, blood stream infection, and health care utilization, even among patients whose culture results remain MDRO positive.^4,5,6,11^ Two study participants in this study died of unrelated conditions and 1 patient was treated with antibiotics when transferred for acute respiratory events. However, these data show numerically reduced days of antibiotic therapy rates and fewer bacteremia episodes among FMT recipients in the 6 months after screening. Ascertainment of these outcomes may have been less complete for controls than FMT recipients due to the potential for receiving care outside of health care systems with available medical record access. However, if infections and antibiotic treatment for controls were more frequent compared with FMT recipients than recorded, the effect of this imbalanced ascertainment would be expected to be a bias toward the null. Taken together, these data support a model of clinical benefit pathogen burden reduction, even among those whose culture results remain positive for MRDO colonization. This potential benefit would require larger cohorts for formal hypothesis testing.

### Limitations

This study had notable limitations. FMT-treated participants and controls were not randomized, and treatment allocation was not concealed. We were unable to match by baseline MDRO category, pathogen relative abundance, or length of stay, which may provide more meaningful comparisons in future studies of similar cohorts. Although 1 participant vomited after gastrostomy instillation, this route of administration is likely still acceptable for many patients, particularly if volumes less than 250 mL are administered. Response was assessed by qualitative culture methods with positive or negative results, thus potential quantitative reductions in pathogen density were not measured in this study. LTACH patients experience competing risks of mortality (20% in this study) and frequent empiric antibiotic exposure given tenuous clinical status, as well as colonization pressure that increases the potential for new MDRO colonization after FMT. These competing risks would all be expected to bias outcome measures toward the null hypothesis of no effect of FMT. Progression from colonization to infection is a relatively rare event in many populations, making this a challenging end point to measure. Studying interventions in populations with a higher colonization prevalence and incidence of progression to infection increases the efficiency of measuring these important end points but also potentially increases the frequency of competing risks with many safety and efficacy outcomes. These limitations are offset by demonstration of feasibility of screening for microbiome interventions with facilitywide prevalence sampling followed by targeted treatment.

## Conclusions

This open-label nonrandomized clinical trial found that FMT was well tolerated and feasible to administer in a high–MDRO prevalence setting. Future studies should optimize microbiota conditioning and dosing strategies to identify approaches to increase efficacy for decolonization and determine effects of microbiota therapies on MDRO transmission. Although a small cohort was studied, these findings suggest that microbiota therapies, particularly with improved formulations that are easier to administer and acceptable to patients, could have potential for preventive treatment for patients at high risk of infection or transmission. This approach could be especially important for LTACHs and other health care facilities that care for patients with a high prevalence of intestinal MDRO colonization for which there are no currently available FDA-approved therapies.

## Notes

Supplement 1.
Trial Protocol and Statistical Analysis Plan

Supplement 2.
eMethods.
**eTable 1.** Serious adverse events stratified at participant and event level by body system and relatedness to fecal microbiota transplant product or procedures. **eTable 2.** Solicited adverse events among fecal microbiota transplant (FMT) recipients **eTable 3.** Crude difference in antibiotic days of therapy rate per 1000 patient-days between fecal microbiota transplant recipients and untreated multidrug-resistant organism–positive contemporaneous controls over the 6 months before and after prevalence sampling **eTable 4.** Difference in antibiotic days of therapy rate per 1000 patient-days between fecal microbiota transplant recipients and untreated contemporaneous controls over the 6 months before and after prevalence sampling using a difference-in-differences model **eFigure 1.** Overall multidrug-resistant organism prevalence among patients admitted to a long-term acute care hospital in 2023 in 2 prevalence surveys 35 days apart to screen for eligibility for the Sentinel REACT trial **eFigure 2.** Summary of respiratory culture isolate count and identification in the 6 months before and after prevalence sampling stratified by fecal microbiota transplantation receipt vs untreated contemporaneous controls **eFigure 3.** Summary of urine culture isolate count and identification in the 6 months before and after prevalence sampling stratified by fecal microbiota transplantation receipt vs untreated contemporaneous controls **eFigure 4.** Summary of positive clinical microbiology cultures per participant among fecal microbiota transplantation recipients vs untreated multidrug-resistant organism–positive contemporaneous controls in 6 months before compared to 6 months after prevalence survey sampling by participant and specimen type
eReferences.

Supplement 3.
Data Sharing Statement
