# Supplementary material for: Microbiota Transplantation Among Patients Receiving Long-Term Care: The Sentinel REACT Nonrandomized Clinical Trial
Source: JAMA Netw Open. 2025 Jul 24;8(7):e2522740. doi: 10.1001/jamanetworkopen.2025.22740 (PMC12290730; doi:10.1001/jamanetworkopen.2025.22740)
Supplement: Supplement 1. — Trial Protocol and Statistical Analysis Plan [file jamanetwopen-e2522740-s001.pdf]

PROTOCOL TITLE: Sentinel Cohort for the Response to Emerging Antimicrobial resistance with Containment microbiota restoration Therapy Trial (Sentinel REACT)

SPONSOR/INVESTIGATOR:

Michael H. Woodworth, MD, MSc

Department of Infectious Disease

404-712-8889

michael.holmes.woodworth@emory.edu

VERSION:

01NOV2024

FUNDING SOURCE:

U.S. Centers for Disease Control & Prevention (CDC) - Prevention Epicenters Program (PEACH II)

REVISION HISTORY

Revision #	Version Date	Summary of Changes
0	17 Feb 2023	Initial Protocol Version
1	20 Mar 2023	Changes:<br>1. Included the IRB requested exclusion criterion for those at risk of bleeding with hemorrhoids.<br>2. Clarified the control Groups A and B as the informed consents were split for Group FMT, Group A and Group B.
2	05 Apr 2023	Modifications:<br>1. Correction of Table number for Appendix A Table 6 mentioned on Page 12.<br>2. Study Schema image modified to include Day 21.<br>3. Modification of FMT administration details.<br>4. Removal of NPO status in FMT administration details.<br>5. Inclusion of solicited AE monitoring for FMT administration group on Day 1, 2, 3, 4, 5, and 6.<br>6. Inclusion of +/-3 day window for Days 7, 14, 21, 28.<br>7. Inclusion of Day 21 in the day to day description between Day 14 and Day 28.<br>8. Inclusion of statement in the day to day description for patients that are discharged before Day 7,14,21,28 to collect and send samples from home.<br>9. Inclusion of Appendix C detailing instructions for patients that are discharged before Day 7,14,21,28 to collect and send samples from home.<br>10. Removal of statement under Day 14 for requiring swabs to be negative on Day 7 to perform MDRO analysis.<br>11. Removal of specific Off-Treatment Criteria.<br>12. Modification of Follow-Up Assessment from Solicited AEs to Serious AEs and AESIs.<br>13. Removal of mention of unblinded epidemiologist sending reports under Measures to Minimize Bias<br>14. Modification of Safety Assessments to include more details.

		15. Modification of inclusion criteria regarding discontinuing antibiotics etc up to Day 28.<br>16. Addition of inclusion criterion regarding patients willing to undergo hCG testing on FMT day.<br>17. Expansion of details on exclusion criterion on uncontrolled intercurrent illnesses.<br>18. Addition of mention of compensation for control Group A participants.<br>19. Modification of Safety Review Triggers to include specific details.<br>20. Modification of Schedule of Events Tables for participants in Group FMT, Group A and Group B.<br>21. Other minor modifications for formatting etc.
3	4 Dec 2023	Modifications:<br><br>1. Addition of chart review of Sentinel REACT participants and matched controls admitted to the same facility to estimate frequency of clinical infection after FMT.
4	1 Nov 2024	Modifications:<br><br>1. Remove Ahmed Babiker as Sub-I, moved to new institution.<br>2.

Table of Contents

1. STUDY SUMMARY 7
2. OBJECTIVES 11
3. BACKGROUND 11
4. STUDY ENDPOINTS 12
4.1. PRIMARY ENDPOINT 13
4.2. SECONDARY ENDPOINTS 13
4.3. EXPLORATORY ENDPOINTS 13
5. STUDY INTERVENTION/INVESTIGATIONAL AGENT 14
6. PROCEDURES INVOLVED 14
6.1. STUDY DESIGN 14
6.2. STUDY SCHEMA 16
6.3. DOSING AND ADMINISTRATION 16
6.4. POST-INTERVENTION ADMINISTRATION OBSERVATION PERIOD 17
6.5. PREPARATION/HANDLING/STORAGE/ACCOUNTABILITY 18
7.1. DETAILED STUDY VISIT (LOCATION) DESCRIPTIONS 21
7.2. MEASURES TO MINIMIZE BIAS: RANDOMIZATION AND BLINDING 26
7.3. STUDY INTERVENTION COMPLIANCE 27
7.4. CONCOMITANT THERAPY 27
7.5. RESCUE MEDICINE 27
8. STUDY ASSESSMENTS AND PROCEDURES 27
8.1. POPULATION AND PROCEDURES: 27
8.2. EFFICACY ASSESSMENTS 28
8.3. SAFETY ASSESSMENTS 29
9. STATISTICAL ANALYSIS PLAN 30
9.1. GENERAL APPROACH 30
9.2. PLANNED INTERIM ANALYSES 30
10. DATA AND/OR SPECIMEN BANKING 30
10.1. FUTURE USE OF STORED SPECIMENS AND DATA 30
11. SHARING OF RESULTS WITH PARTICIPANTS 31
12. STUDY TIMELINES 32
12.1. END OF STUDY DEFINITION 32
13. INCLUSION AND EXCLUSION CRITERIA 32

13.1. STUDY POPULATION 32
13.2. HUMAN SUBJECTS RESEARCH REVIEW 32
13.3. INCLUSION CRITERIA 33
13.4. EXCLUSION CRITERIA 34
13.5. LIFESTYLE CONSIDERATIONS 35
14. VULNERABLE POPULATIONS 35
15. LOCAL NUMBER OF PARTICIPANTS 35
16. RECRUITMENT METHODS 35
16.1. STRATEGIES FOR RECRUITMENT AND RETENTION 35
17. WITHDRAWAL OF PARTICIPANTS 35
17.1. DISCONTINUATION/WITHDRAWAL OF STUDY INTERVENTION 35
17.2. PARTICIPANT DISCONTINUATION/WITHDRAWAL FROM THE STUDY 36
17.3. LOST TO FOLLOW-UP 36
17.4. OFF-STUDY CRITERIA 37
18. RISK TO PARTICIPANTS 37
18.1. KNOWN POTENTIAL RISKS 37
18.2. LONG-TERM IP RISKS 40
18.3. PROCEDURAL RISKS 40
18.4. REPRODUCTIVE RISKS: 41
18.5. NON-PHYSICAL RISKS: 41
19. POTENTIAL BENEFITS TO PARTICIPANTS 42
19.1. IMMEDIATE POTENTIAL IP BENEFITS 42
19.2. LONG-RANGE POTENTIAL IP BENEFITS 42
20. COMPENSATION TO PARTICIPANTS 43
21. DATA MANAGEMENT AND CONFIDENTIALITY 43
21.1. CONFIDENTIALITY AND PRIVACY 43
21.2. CERTIFICATE OF CONFIDENTIALITY 45
22. PLANS TO MONITOR THE DATA TO ENSURE SAFETY OF PARTICIPANTS AND DATA INTEGRITY 45
23. MONITORING TABLE 2 47
23.1. CONFLICT OF INTEREST POLICY 51
23.2. SAFETY OVERSIGHT/ DATA SAFETY AND MONITORING BOARD (DSMB) 51
23.3. QUALITY ASSURANCE AND QUALITY CONTROL 52
23.4. DATA HANDLING AND RECORD KEEPING 52
23.5. ADVERSE EVENTS AND SERIOUS ADVERSE EVENTS 54

23.6. UNANTICIPATED PROBLEMS 59
23.7. SPONSOR-INVESTIGATOR REQUIREMENTS 60
23.8. EXPEDITED REPORTING 60
23.9. HALTING RULES 60
23.10. SAFETY REVIEW TRIGGERS 61
24. PROVISIONS TO PROTECT THE PRIVACY INTEREST OF PARTICIPANTS 61
25. ECONOMIC BURDEN TO PARTICIPANTS 62
26. INFORMED CONSENT 62
26.1. CONSENT/ASSENT AND OTHER INFORMATIONAL DOCUMENTS PROVIDED TO PARTICIPANTS 62
26.2. REGISTRATION PROCESS 63
26.3. REGISTRATION PROCEDURE DESCRIPTION 64
26.4. NON-ENGLISH-SPEAKING PARTICIPANTS 64
26.5. ADULTS UNABLE TO CONSENT 64
27. SETTING 64
28. RESOURCES AVAILABLE 65
29. REFERENCES 66

1. Study Summary

**Project Title**	Sentinel Cohort for the Response to Emerging Antimicrobial resistance with Containment microbiota restoration Therapy Trial (Sentinel REACT)
**Project Design**	This protocol describes an open label sentinel cohort study of microbiota transplantation (MT) treatment of 10-20 participants who are admitted to a long-term acute care hospital (LTACH) or ventilator-capable skilled nursing facility (vSNF) and colonized by a target multi-drug resistant organism (MDRO) as detected by peri-rectal or stool culture. Safety data from this sentinel cohort were requested by the FDA in advance of a larger multi-center study called REACT. This study is conducted in two parts. In part 1 (the APPS protocol, IRB00004199) facilities undergo periodic point prevalence sampling for qualitative detection of patient multi-drug resistant organism (MDRO) colonization with culture-based assays. In part 2 (the present protocol) all MDRO-positive patients at a participating facility will be offered Microbiome Therapy (MT) for MDRO decolonization with safety and efficacy follow up.
**Primary Objectives**	The primary objective of Sentinel REACT is to evaluate the safety of MT for MDRO decolonization in patients admitted to LTACHs or vSNFs.
**Secondary Objective**	The secondary objective of REACT is to estimate the efficacy of MT delivered via feeding tube for MDRO decolonization.
**Exploratory Objectives**	The exploratory objectives of REACT are to:<br>• Estimate the potential effect size of MT in reducing facility MDRO prevalence.<br>• Evaluate feasibility of participant recruitment in LTACHs and/or vSNFs.<br>• Estimate the potential effect size of MT in reducing facility *C. difficile* infection (CDI) incidence rates.<br>• Estimate the potential effect size of MT in

	reducing facility new MDRO colonization incidence rates at 30 days.<br>• Estimate the potential effect size of MT in reducing facility MDRO blood stream infection (BSI) incidence rates.<br>• Compare frequency of any infection (bacterial, fungal, viral) within six months prior and six months post treatment in MT-treated participants versus contemporaneous controls.<br>• Compare frequency of MDRO infection within six months prior and six months post treatment in MT-treated participants versus contemporaneous controls.<br>• Identify AR genes with the greatest log-fold increase and decrease in patients treated with MT.<br>• Identify presumptive donor-derived strains that are most frequently detected in MT recipients prior to MDRO decolonization.<br>• Identify presumptive donor-derived genes that are most frequently detected in MT recipients prior to MDRO decolonization.<br>• Determine engraftment dynamics of likely donor-derived strains in MT treated recipients.<br>• Identify differences in MDRO decolonization efficacy by MT manufacturing lot.<br>• Identify differences in MDRO decolonization efficacy by MT donor.<br>• Identify potential clusters and pathways of MDRO transmission.
**Research Intervention(s)/Interactions**	**Microbiome Therapeutic (MT)**: Participants will receive an Emory-manufactured MT product, delivered as 250mL via an existing feeding tube or rectal enema (if a functioning feeding tube is not in place) with the rate adjusted to recipient tolerance.
**Study Population**	Patients admitted to long-term care facilities (i.e. long-term acute care hospitals [LTACH], ventilator-capable skilled nursing facilities [vSNF], and tracheostomy care units [TCU]) at Emory Long Term Acute Care (ELTAC).
**Sample Size**	Locally: Up to 75 participants from Emory Long-Term Acute Care [ELTAC] will be enrolled.

	The target enrollment for this study is 1-2 facilities with a goal to treat 10-20 MDRO-positive participants with MT.<br><br>It is anticipated that this study will run for one year.
**Study Duration for individual participants**	Approximately 4 weeks of sample collection and AE monitoring, followed by 24 weeks (6 months) of monthly AE and questionnaire follow up by telephone and/or email.
**Study Specific Abbreviations/ Definitions**	• AE: Adverse Event<br>• ANC: Absolute Neutrophil Count<br>• AR: Antimicrobial Resistance<br>• ASA: American Society of Anesthesiologists<br>• BSI: Bloodstream Infection<br>• cART: Combination Antiretroviral Therapy<br>• CBER: Center for Biologics Evaluation and Research (part of FDA)<br>• CDI: *Clostridioides difficile* (*C. difficile*) Infection<br>• CFR: Code of Federal Regulations<br>• CLIA: Clinical Laboratory Improvement Amendments<br>• CMV: Cytomegalovirus<br>• Co-I: Co-Investigator<br>• CRAB: Carbapenem-resistant *Acinetobacter baumanii*<br>• CRE: Carbapenem-resistant *Enterobacteriaceae*<br>• CRF: Case Report Form<br>• CRPA: Carbapenem-resistant *Pseudomonas aeruginosa*<br>• CTCAE: Common Terminology Criteria for Adverse Events (v5.0)<br>• DFA: Direct Fluorescent Antibody<br>• DSMB: Data and Safety Monitoring Board<br>• EAEC: Enteroaggregative *Escherichia coli*<br>• EDC: Electronic Data Capture<br>• EPEC: Enteropathogenic *Escherichia coli*<br>• ERMS: Emory Research Management System<br>• ESBL: Extended Spectrum Beta-Lactamase<br>• EML: Emory Medical Laboratory<br>• ETEC: Enterotoxigenic *Escherichia coli*<br>• FDA: Food and Drug Administration

	• FMT: Fecal Microbiota Transplantation<br>• GCRC: Georgia CTSA Clinical Research Centers<br>• GX®: Cepheid GeneXpert ®<br>• hCG: Human chorionic gonadotropin<br>• HIPAA: Health Insurance Portability and Accountability Act (of 1996)<br>• HIV: Human Immunodeficiency Virus<br>• IBD: Inflammatory Bowel Disease<br>• IBS: Irritable Bowel Syndrome<br>• ICF: Informed Consent Form<br>• IHC: Immunohistochemical<br>• IND: Investigational New Drug<br>• IP: Investigational Product<br>• IRB: Institutional Review Board<br>• LTACH: Long-Term Acute Care Hospital<br>• MDR: Multi-Drug Resistant<br>• MDRO: Multi-Drug Resistant Organism<br>• MT: Microbiota Therapy<br>• NGS: Next-Generation Sequencing<br>• OCR: Emory Office for Clinical Research<br>• PCR: Polymerase Chain Reaction<br>• PPI: Proton Pump Inhibitor<br>• PRN: As Needed<br>• RCDI: Recurrent *Clostridioides difficile* Infection<br>• SAE: Serious Adverse Event<br>• SI: Sponsor-Investigator<br>• SOT: Solid Organ Transplant<br>• Sub-I: Sub-Investigator<br>• TCU: Tracheostomy Care Unit<br>• VACC: Vancomycin Amphotericin B, Ceftazidime, And Clindamycin Agar<br>• VIM: Verona Integron-Mediated Metallo-β-lactamase<br>• VRE: Vancomycin-Resistant *Enterococcus*<br>• WHOQOL: World Health Organization Quality of Life survey<br>• WOCBP: Women of Child-Bearing Potential
**Funding Source (if any)**	U.S. Centers for Disease Control & Prevention (CDC) - Prevention Epicenters Program (PEACH II)<br><br>Source: U54CK000601

2. Objectives

The primary objective of Sentinel REACT is: to evaluate the safety of MT for MDRO decolonization in patients admitted to LTACHs or vSNFs.

The secondary objective of Sentinel REACT is: to estimate the efficacy of MT delivered via feeding tube for MDRO decolonization.

The exploratory objectives of Sentinel REACT are to:

- Estimate the potential effect size of MT in reducing facility MDRO prevalence.
- Evaluate feasibility of participant recruitment in LTACHs and/or vSNFs.
- Estimate the potential effect size of MT in reducing facility *C. difficile* infection (CDI) incidence rates.
- Estimate the potential effect size of MT in reducing facility MDRO prevalence.
- Evaluate feasibility of participant recruitment in LTACHs and/or vSNFs.
- Estimate the potential effect size of MT in reducing facility *C. difficile* infection (CDI) incidence rates.
- Estimate the potential effect size of MT in reducing facility new MDRO colonization incidence rates at 30 days.
- Estimate the potential effect size of MT in reducing facility MDRO blood stream infection (BSI) incidence rates.
- Compare frequency of any infection (bacterial, fungal, viral) within six months prior and six months post treatment in MT-treated participants versus contemporaneous controls.
- Compare frequency of MDRO infection within six months prior and six months post treatment in MT-treated participants versus contemporaneous controls.
- Identify AR genes with the greatest log-fold increase and decrease in patients treated with MT.
- Identify presumptive donor-derived strains that are most frequently detected in MT recipients prior to MDRO decolonization.
- Identify presumptive donor-derived genes that are most frequently detected in MT recipients prior to MDRO decolonization.
- Determine engraftment dynamics of likely donor-derived strains in MT treated recipients.
- Identify differences in MDRO decolonization efficacy by MT manufacturing lot.
- Identify differences in MDRO decolonization efficacy by MT donor.
- Identify potential clusters and pathways of MDRO transmission with genomic, metagenomic, location, and provider badge proximity data.

3. Background

The emergence of novel antimicrobial resistance (AR) mechanisms presents major threats to

public health.[1] When encoded on mobile genetic elements, these AR determinants can disseminate throughout populations increasing morbidity, mortality, and cost given limited treatment options. Isolates with resistance to all clinically-available antibiotics are increasingly identified[2] Although the response to emerging multi-drug resistant organisms (MDRO) such as carbapenem-resistant Enterobacteriaceae (CRE) have shown limited success[3], many approaches are less effective once MDROs become prevalent. Multi-drug resistant organism (MDRO) colonization represents a crucial opportunity for intervention because it frequently precedes potentially fatal infections.[4,5] In addition, colonization promotes transmission to other patients. MDRO colonization thus increases direct healthcare costs of MDRO-related health impacts and increases indirect costs of CMS penalties in high-prevalence healthcare facilities. Unfortunately, there are no approved therapies for intestinal decolonization.

The intestinal tract is a major reservoir of many enteric MDROs like CRE and extended-spectrum β-lactamase producing Enterobacteriaceae (ESBL-E). This reservoir is increasingly recognized as an important therapeutic target to reduce asymptomatic patient MDRO colonization with microbiome therapeutics (MT). Many MT benefits are analogous to vaccination, including reducing risk of infection for an individual and thereby potentially reducing asymptomatic shedding and transmission to susceptible patients. MT such as fecal microbiota transplantation (FMT) are up to 90% efficacious for reducing culture-detected colonization with multi-drug resistant organisms (MDROs) but they have not yet been evaluated in long-term care facilities. This is problematic because long-term care facilities are recognized to have a higher burden of MDRO prevalence than other care settings.[6]

The status quo of AR response is focused on case finding and bundled enhanced barrier precautions containment measures.[7] These methods can reduce transmission but have limited effectiveness in reducing patient colonization once established. Prior work has established that individual patients can have disproportionate contributions to MDRO contamination of healthcare environments and serve as vectors to transmit MDROs between multiple healthcare facilities.[8] Retrospective studies of patients with RCDI treated with FMT have indicated that FMT may reduce blood stream infections and mortality compared to standard of care.[9,10] Reduced MDRO infection has also been demonstrated in a small, non-randomized observational study of 20 MDRO-colonized patients, FMT had significant reductions in antibiotic treatment, bacteremia, and length of stay.[11] Although FMT has been shown in small studies to be 38-88% effective in patient MDRO elimination, many of these studies lacked control groups, longitudinal follow-up, or genomic strain tracking. Thus, there are many potential benefits of FMT for MDRO colonization that that warrant further study in controlled, prospective clinical trials. Further, the potential to indirectly reduce population transmission from MDRO-colonized patients is a plausible potential population-level benefit of this patient-level intervention that has not been studied.

4. Study Endpoints

4.1. Primary Endpoint

1) The safety of MT in this population with a high burden of comorbidities will be estimated by:
    a. Frequency and severity of AEs at Day 7.
    b. Frequency and severity of solicited AEs from Day 0 until Day 196 (Day 28 + 6 months)

4.2. Secondary Endpoints

2) MT MDRO decolonization efficacy will be estimated by:
    a. Proportion of MT-recipient stool cultures at Day 14 positive for any target MDRO.

4.3. Exploratory Endpoints

Study IDs from REACT will be linked to the companion periodic prevalence sampling protocol APPS (IRB00004199) to evaluate:

1) Facility cumulative and category-specific MDRO prevalence at Day 28 compared to baseline.
2) Average facility *C. difficile* infection (CDI) incidence rate two months before and two months after MT.
3) Average facility CRE positive clinical culture incidence rates two months before and two months after MT.
4) Average facility blood stream infection (BSI) incidence rates two months before and two months after MT.
5) Average facility central line-associated blood stream infection (CLABSI) incidence rates two months before and two months after MT.
6) Average facility catheter-associated urinary tract infection (CAUTI) incidence rates two months before and two months after MT.
7) Frequency of infection (bacterial, fungal, viral) six months before and six months after MT in MT treated participants compared to contemporaneous controls who were admitted at time of prevalence sampling but not treated with MT.
8) Frequency of MDRO infection six months before and six months after MT in MT treated participants compared to contemporaneous controls who were admitted at time of prevalence sampling but not treated with MT.
9) Log-fold change in AR gene peri-rectal and inguinal metagenomic coverage from selected patients treated with MT.
10) Differential detection and abundance tests of likely donor-derived strains in MT recipient peri-rectal swab metagenomes.
11) Tests of metagenomic coverage breadth trends for likely donor-derived strains in MT-treated recipients.
12) Tests of metagenomic coverage breadth trends for likely donor-derived genes in MT treated recipients.

13) Difference in proportion of MDRO decolonization by culture between recipients administered MT vs eligible patients who declined MT and enrolled in the control groups.
14) Difference in SAEs at Day 7 between recipients administered MT vs eligible patients who declined MT and enrolled in the control groups.
15) Difference in SAEs at Day 28 between recipients administered MT vs eligible patients who declined MT and enrolled in the control groups.
16) Difference in proportion of MDRO decolonization by culture between recipients administered MT via feeding tube vs enema (if enema treated participants n > 1).
17) Difference in SAEs at Day 28 between recipients administered MT via feeding tube vs enema (if enema treated participants n > 1).
18) Isolate genome cluster analyses and measures of similarity (e.g. average nucleotide identity, counts of single nucleotide variants) layered with room and provider location data to identify potential transmission clusters.

See **Appendix A** for **Table 6** which consolidates the objectives, endpoints and the endpoint justifications.

5. Study Intervention/Investigational Agent

Description: The investigational MT is manufactured with a range of 50-100mg of stool from highly screened donors (as described in the MEP stool donor protocol IRB00112302) suspended in 250mL USP sterile 0.9% saline using a benchtop stomacher device, combined with 10% glycerol by volume and stored in labeled 250mL saline bottles.

Drug Handling: After manufacturing in Woodruff Memorial Building 7006 under GMP, the MT is stored at -80 degrees Celsius and access is restricted by a hallway keycard and lab door key or keypad. The MT is thawed when ordered by study staff, and instilled by study staff via enteric feeding tube (preferred when functional tube is in place) or as a retention enema. The investigational MT is not approved as a commercially available product. The MT will only be released to authorized, trained investigators and only administered to study participants after informed consent has been provided by the participant or their legally authorized representative. The sponsor-investigator Dr. Michael Woodworth will hold the IND for this study.

6. Procedures Involved

6.1. Study Design

Sentinel REACT is a phase 1, open-label observational study to collect safety data for the use of MT in patients admitted to participating LTACHs, vSNFs, and TCUs with intestinal MDRO

colonization detected by peri-rectal or stool culture. The IP is manufactured from stool collected from highly screened donors. Donor stool is suspended in normal saline and 10% glycerol and frozen at -80 degrees Celsius until use. The goal of Sentinel REACT is to enroll 10-20 participants with positive MDRO cultures from the APPS study (IRB00004199) for periodic peri-rectal and inguinal sampling of MDRO (*C. difficile,* CRE, CRPA, ESBL, and VRE) prevalence. Safety data including frequency and severity of AEs of MT-treated participants at Day 7 will be summarized and presented to the DSMB for review after a minimum of 10 but no more than 20 participants have been treated. Efficacy and exploratory analyses may be prepared for presentation or publication or used to refine power estimates and optimize study design for REACT.

Eligible participants can consent to take part in one of three ways:

1. Consent to receive MT and provide stool/peri-rectal swab samples and medical record review. (Group FMT)
2. Consent to only provide stool/peri-rectal swab samples and medical record review and decline MT. (control Group A)
3. Consent to only allow medical record review and decline MT and swab samples. (control Group B)

6.2. Study Schema

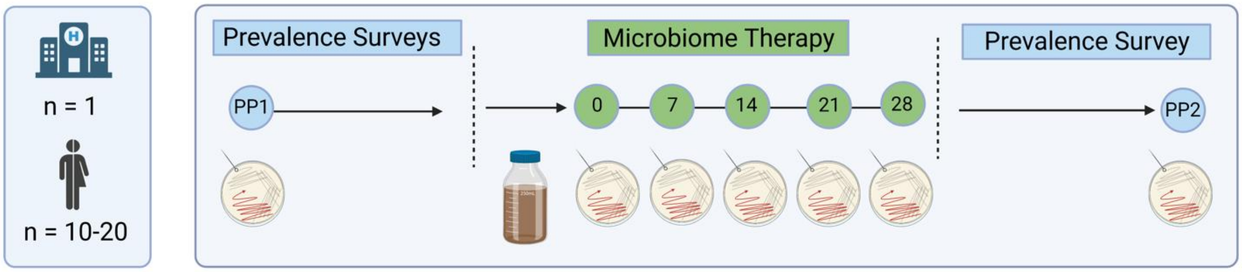

Fig 6.2.1: Study schema for MDRO prevalence sampling of facilities participating in the APPS study (ELTAC) one month before and after MT. Up to 20 MDRO-positive patients identified from the APPS study will be approached to discuss potential participation in Sentinel REACT and obtain informed consent for MT. After MT, participants undergo peri-rectal and inguinal swabs and stool collection when feasible on days 0, 7, 14, 21 and 28. Participants who are discharged prior to final scheduled sample collection will continue safety follow up by telephone visits but censored for microbiology and metagenomic analyses.

6.3. Dosing and Administration

MT will be delivered to participants as 250mL instilled via functional feeding tube when in place or rectal enema (when a functional feeding tube is not present) with the rate adjusted to the recipient's clinical status and infusion tolerance.

6.3.1. Intervention Administration Instructions (Feeding Tube, Bolus Instillation)

1. Complete order for treatment dose from Georgia CTSA Clinical Research Center (GCRC) biorepository.
2. Study staff thaw treatment doses.
3. Study staff pack doses for treatment in cooler for transport to facility.
4. Maintain NPO status for approximately two hours prior to IP administration.
5. IP (250mL) is instilled via feeding tube **using a 60cc syringe over 15 mins**.
6. Resume prior activity as ordered 60 minutes after IP administration if no adverse events are observed.
7. Resume prior diet as ordered 2 hours after IP administration if no adverse events are observed.

6.3.2. Intervention Administration Instructions (Feeding Tube, Pump Instillation)

1. Complete order for treatment dose from Georgia CTSA Clinical Research Center (GCRC) biorepository.
2. Study staff thaw treatment doses.
3. Study staff pack doses for treatment in cooler for transport to facility.
4. Maintain NPO status for approximately two hours prior to IP administration.
5. IP (250mL) is poured into hung tube feed bag and instilled via feeding tube **using a**

programmable pump at 1000mL/hour (or 250mL over 15 mins).

6. Resume prior activity as ordered 60 minutes after IP administration if no adverse events are observed.
7. Resume prior diet as ordered one hour after IP administration if no adverse events are observed.

6.3.3. Intervention Administration Instructions (Enema)

1. Complete order for treatment dose from Georgia CTSA Clinical Research Center (GCRC) biorepository.
2. Study staff thaw treatment doses.
3. Study staff pack doses for treatment in cooler for transport to facility.
4. Patients who do not have a history of constipation may be pre-medicated with (loperamide) to help retain the enema.
5. The IP will be allowed to flow through the tubing to minimize air infused into the rectum.
6. Position participant’s body (pre-intervention) supinely as follows:
   - Head on a pillow
   - Buttocks on a thick absorbent pad or towel
   - Knees bent
7. Affix enema bag to a hook 12 inches above the participant
8. Collect Day 1 perirectal swabs if stool not provided
9. Insert the lubricated enema tip 3-4 inches into the rectum (length of index finger)
10. Position participant’s body in the Trendelenburg position if possible with both feet approximately 10 inches higher than the head
11. Infuse IP at a rate tolerable to the participant
12. During the first 30 minutes (as tolerated), participant’s abdomen should be massaged in a counter-clockwise direction (slowly and deeply)
    Note: this is to facilitate movement of the IP in the colon and can be performed by the person administering the enema or the participant. If the participant experiences cramping, temporarily hold the enema infusion and advise the participant to take quick, shallow breaths. When discomfort passes, resume the enema.
13. Upon completion of administration, the tube is clamped off and the nozzle removed.
14. Resume prior activity as ordered 60 minutes after IP administration if no adverse events are observed.
15. Resume prior diet as ordered 2 hours after IP administration if no adverse events are observed.

6.4. Post-Intervention Administration Observation Period

Participants will be observed for at least 30 minutes after the procedure to ensure tolerance of IP administration and record and address any potential AEs.

6.5. Preparation/Handling/Storage/Accountability

6.5.1. Acquisition and Accountability

The study IP will be provided to the investigator by the GCRC biorepository staff after an order is placed. The GCRC biorepository will maintain accountability logs of product and maintain a record of all allocated treatment. Expired or unused IP will be destroyed or assigned to research use after approval by the Sponsor-Investigator.

6.5.2. Temperature Monitoring

To ensure that investigational drugs are stored at the appropriate temperature, the GCRC biorepository uses a continual temperature monitoring system. Sensors are placed to monitor freezer temperatures.

6.5.3. Restricted Access

Only GCRC biorepository personnel carry keys and have routine access to GCRC biorepository locations.

6.5.4. Formulation, Appearance, and Labeling

6.5.4.1. Formulation

The IP is manufactured using human stool from a healthy stool donor participant under Emory IRB protocol (IRB00112302). Human stool specimens are heterogeneous substances, composed of bacteria, viral, and fungal organisms, metabolic products of these organisms, undigested foods, dead cells, and mucus from the lining of the intestinal wall. The exact composition of human stool varies from donor to donor and from day to day. In effort to standardize the composition for this clinical trial, all the IP will be from as few donors as possible. The IP will be prepared as a suspension to facilitate instillation via feeding tube or enema. Given the natural variation of human stool composition, some of the physical characteristics will be unknown. IP manufacturing includes suspension of 50-100g of stool in 250mL sterile normal saline USP using a benchtop stomacher, which is then stored in the original saline bottle with 10% glycerol by volume (approximately 25mL glycerol / 250mL fecal suspension) and frozen at -80 °C in a freezer in a locked laboratory with continuous temperature monitoring.

6.5.4.2. Appearance

The IP appears as a brown, opaque liquid (solid when frozen) in a 250mL saline bottle labeled as described elsewhere per IND specifications.

6.5.4.3. Packaging and Labeling

The bottles of IP will be labeled with the protocol acronym, manufacturer (Sponsor-Investigator), address and phone number, IP name, lot and bottle numbers, as well as the dates of manufacturing and expiration.

Additionally, each bottle will include the following warnings:

- "Caution: New Drug--Limited by Federal (or United States) law to investigational use"
- Indication for enteral use only

Example initial **NON-RELEASED PRODUCT** label:

NON-RELEASED PRODUCT		
**INVESTIGATIONAL PRODUCT FOR ENTERAL USE ONLY.**<br>*IP expires 24 months after stool sample processing.*		
**CAUTION**: New Drug –<br>Limited by Federal (or United States) law to investigational use.		
**Protocol**: REACT	**IRB #:** ***	**IND #** : ***
**IP**: Allogeneic Microbiota in Glycerol (10%) (AMG)		
**Sponsor-Investigator/Manufacturer:** Michael H. Woodworth, MD, MSc, Emory University, 1760 Haygood Dr NE, Atlanta, GA 30322 (Phone: +1-404-234-8452)		
Date of manufacturing:		
Date of expiry:		
Tracking Numbers:	Lot #:	Bottle ____ of ____

After repeat blood, urine, and stool testing is performed without any results that would warrant lot destruction, the material manufactured in the interval between negative testing points will be overlabeled with the following **RELEASED PRODUCT** label:

RELEASED PRODUCT		
**INVESTIGATIONAL PRODUCT FOR ENTERAL USE ONLY.**<br>*IP expires 24 months after stool sample processing.*		
**CAUTION**: New Drug –<br>Limited by Federal (or United States) law to investigational use.		
**Protocol**: REACT	**IRB #:** ***	**IND #** : ***
**IP**: Allogeneic Microbiota in Glycerol (10%) (AMG)		
**Sponsor-Investigator/Manufacturer:** Michael H. Woodworth, MD, MSc, Emory University, 1760 Haygood Dr NE, Atlanta, GA 30322 (Phone: +1-404-234-8452)		

6.5.5. Placebo description

There is no placebo for this study.

6.5.6. Product Storage and Stability

The IP will be stored under appropriate conditions in accordance with the study-specific labels, in a secure location with restricted access. The storage conditions will be monitored regularly, and the temperature will be documented in standard laboratory SOPs.

7.1. Detailed Study Visit (location) Descriptions

A detailed Schedule of Events appears as Appendix A (Table 6).

7.1.1 Screening (hospital room):

Sentinel REACT participants are identified from periodic prevalence sampling performed under the APPS protocol. All MDRO-positive patients will be linked back to a local patient roster dataset with personal identifiers to facilitate linking to approach patients. Patient medical records will be reviewed and their clinical course discussed with providers to evaluate eligibility. The screening tests below may show that an individual who screened positive for a target MDRO during APPS but is not eligible to participate in the research study. However, if someone meets the eligibility criteria, and either they or their LAR provide informed consent, they will be given a study calendar with information about what to expect during and between study visits.

During screening, a trained study team member will obtain and/or conduct the following:

- **Medical history:** A participant's medical record will be reviewed to collect information about their demographic characteristics (i.e. age, sex, race, ethnicity), clinical characteristics (i.e. medical history, current or anticipated antibiotic prescriptions), prior surgeries, and allergies. If someone is currently taking systemic antibiotics for treatment of an infection or if they are taking preventative antibiotics, these will need to be completed at least 2 days before their first MT.
- **Physical exam**: Participants will receive a physical exam, assessment of vital signs (blood pressure, heart rate, temperature), and weight.
- **Urine Pregnancy Test:** Women of childbearing potential (i.e. no reported history of menopause, hysterectomy, abstinence, or procedural contraception such as an intrauterine device or tubal ligation) will have a pregnancy test performed. If the test is positive, their participation will be on hold until after they are no longer pregnant or breastfeeding.

7.1.2 Day 0 (hospital room):

- **Adverse event and concomitant medication review**: The study team will review pre-specified solicited AEs on Day 0, 1, 2, 3, 4, 5, 6, and below mentioned Day 7 and so

on. All AEs will be monitored from Day 0 through the end of the study including in the follow-up 6 month period.

- **Microbiome Therapeutic administration (MT)**
  - **Pre-medication (if no feeding tube)**: Participants who do not have a history of constipation or feeding tube in place may be pre-medicated with 4mg loperamide by mouth to help retain the enema.
  - **Microbiome therapeutic (MT) delivery**: Participants will receive 250mL of the MT via an existing feeding tube or enema with the rate adjusted to the participant's tolerance.
  - **Observation period**: After the infusion is complete, participants will be observed by the study team for immediate potential adverse events for at least one hour.
- **MDRO analyses,** which includes the following tests:
  - **Stool sample or perirectal swab culture:** stool or perirectal swab test for MDROs, which involves participants providing a stool sample or a brief insertion of a culture swab approximately 3 cm into the anus. Participants may be informed of the qualitative results of these cultures (i.e. whether any target MDRO was isolated) if they wish. Isolates from these cultures may be stored for up to 10 years.
  - **Stool or perirectal swab metagenomic sequencing**: samples collected from a participant's stool will be sent for metagenomic sequencing to look at all of the types of bacteria, viruses and fungi found in their stool. Participants will not be informed of these results and the results do not affect eligibility for study participation. The samples may be stored for up to 10 years.
  - **Environmental surface sampling**: environmental surface composites (e.g. bedside table, bedrail, phone; toilet seat, bathroom sink drain trap, or bedside commode as applicable; doorhandle, hand hygiene pump) will be sampled from a participant's room for culture and metagenomic sequencing. Participants will not be informed of these results and the results do not affect eligibility for study participation. The samples may be stored for up to 10 years.

Day 1, 2, 3, 4, 5, 6 (hospital room)

- **Adverse event and concomitant medication review**: The study team will review pre-specified solicited AEs, non-solicited AEs, and any changes to their medications.

7.1.3 Day 7 +/- 3 days (hospital room):

- **Adverse event and concomitant medication review**: The study team will review pre-specified solicited AEs, non-solicited AEs, and any changes to their medications.
- **MDRO analyses,** which includes the following tests:
  - **Stool sample or perirectal swab culture:** stool or perirectal swab test for MDROs, which involves participants providing a stool sample or a brief insertion of a culture swab approximately 3 cm into the anus. Participants may be informed of the qualitative results of these cultures (i.e. whether any target

MDRO was isolated) if they wish. Isolates from these cultures may be stored for up to 10 years.

- **Stool or perirectal swab metagenomic sequencing**: samples collected from a participant's stool will be sent for metagenomic sequencing to look at all of the types of bacteria, viruses and fungi found in their stool. Participants will not be informed of these results and the results do not affect eligibility for study participation. The samples may be stored for up to 10 years.
- **Environmental surface sampling**: environmental surface composites (e.g. bedside table, bedrail, phone; toilet seat, bathroom sink drain trap, or bedside commode as applicable; doorhandle, hand hygiene pump) will be sampled from a participant's room for culture and metagenomic sequencing. Participants will not be informed of these results and the results do not affect eligibility for study participation. The samples may be stored for up to 10 years.

7.1.4 Day 14 +/- 3 days (hospital room):

- **Adverse event and concomitant medication review**: The study team will review pre-specified solicited AEs, non-solicited AEs, and any changes to their medications.
- **MDRO analyses,** which includes the following tests:
  - **Stool sample or perirectal swab culture:** stool or perirectal swab test for MDROs, which involves participants providing a stool sample or a brief insertion of a culture swab approximately 3 cm into the anus. Participants may be informed of the qualitative results of these cultures (i.e. whether any target MDRO was isolated) if they wish. Isolates from these cultures may be stored for up to 10 years.
  - **Stool or perirectal swab metagenomic sequencing**: samples collected from a participant's stool will be sent for metagenomic sequencing to look at all of the types of bacteria, viruses and fungi found in their stool. Participants will not be informed of these results and the results do not affect eligibility for study participation. The samples may be stored for up to 10 years.
  - **Environmental surface sampling**: environmental surface composites (e.g. bedside table, bedrail, phone; toilet seat, bathroom sink drain trap, or bedside commode as applicable; doorhandle, hand hygiene pump) will be sampled from a participant's room for culture and metagenomic sequencing. Participants will not be informed of these results and the results do not affect eligibility for study participation. The samples may be stored for up to 10 years.

7.1.5 Day 21 +/- 3 days (hospital room):

- **Adverse event and concomitant medication review**: The study team will review pre-specified solicited AEs, non-solicited AEs, and any changes to their medications.
- **MDRO analyses,** which includes the following tests:
  - **Stool sample or perirectal swab culture:** stool or perirectal swab test for MDROs, which involves participants providing a stool sample or a brief insertion of a culture swab approximately 3 cm into the anus. Participants may be

informed of the qualitative results of these cultures (i.e. whether any target MDRO was isolated) if they wish. Isolates from these cultures may be stored for up to 10 years.

- **Stool or perirectal swab metagenomic sequencing**: samples collected from a participant's stool will be sent for metagenomic sequencing to look at all of the types of bacteria, viruses and fungi found in their stool. Participants will not be informed of these results and the results do not affect eligibility for study participation. The samples may be stored for up to 10 years.
- **Environmental surface sampling**: environmental surface composites (e.g. bedside table, bedrail, phone; toilet seat, bathroom sink drain trap, or bedside commode as applicable; doorhandle, hand hygiene pump) will be sampled from a participant's room for culture and metagenomic sequencing. Participants will not be informed of these results and the results do not affect eligibility for study participation. The samples may be stored for up to 10 years.

7.1.5 Day 28 +/- 3 days (hospital room):

- **Adverse event and concomitant medication review**: The study team will review pre-specified solicited AEs, non-solicited AEs, and any changes to their medications.
- **MDRO analyses,** which includes the following tests:
  - **Stool sample or perirectal swab culture:** stool or perirectal swab test for MDROs, which involves participants providing a stool sample or a brief insertion of a culture swab approximately 3 cm into the anus. Participants may be informed of the qualitative results of these cultures (i.e. whether any target MDRO was isolated) if they wish.
  - **Stool metagenomic sequencing**: samples collected from a participant's stool will be sent for metagenomic sequencing to look at all of the types of bacteria, viruses and fungi found in their stool. Participants will not be informed of these results and the results do not affect eligibility for study participation. The samples may be stored for up to 10 years.
  - **Environmental surface sampling**: environmental surface composites (e.g. bedside table, bedrail, phone; toilet seat, bathroom sink drain trap, or bedside commode as applicable; doorhandle, hand hygiene pump) will be sampled from a participant's room for culture and metagenomic sequencing. Participants will not be informed of these results and the results do not affect eligibility for study participation. The samples may be stored for up to 10 years.

If discharged before any of these timepoints, Day 7, Day 14, Day 21 or Day 28, patients will be asked to collect at home stool samples and peri-rectal swabs on these days and send in. Instructions for patients for collecting home stool samples and peri-rectal swabs are included in Appendix C.

7.1.6 Follow-Up Period (remote visit unless hospitalized):

Participants will be followed monthly for 24 weeks after their last study visit (Day 28) is complete to monitor for unsolicited AEs, concomitant medication changes or additions, and completion of the WHO Quality of Life Survey (WHOQOL). This will be done by sending participants a secure online survey, text message, telephone call, or inpatient bedside visit every 4 weeks for 24 weeks. We will attempt to contact participants three times for each follow-up visit. We may also access a participant's medical records to obtain similar information.

7.1.7 Off-Treatment Criteria

If a participant is removed from the research study before receipt of the IP, the research doctor will explain to the participant or their LAR why they were removed. In addition, as part of the consent process, participants will be told that they can stop participating in the research study at any time, however, the FDA requires that any information collected up to the point of their withdrawal cannot be removed from the study. If a participant decides to stop participating in this research study, they will be encouraged to talk to the research doctor and their primary doctor first.

The date and reason for taking a participant off-treatment must be documented in the case report form (CRF) and in OnCore (for Emory participants). Alternative options will be discussed with the participant (see section 15 for further discussion of options and removal from the study). In the event of unusual or life-threatening complications, treating investigators must immediately notify the Sponsor Investigator, Dr. Michael Woodworth, 404-234-8452.

7.1.8 Follow-Up Entry

Participants will enter the Follow-Up period after either:

1. Completing the Day 28 visit.
2. Discontinuation of treatment for any reason other than target MDRO elimination.

Participants who come off treatment for AE(s) directly related to administration of the IP treatment, will be followed until resolution or stabilization of the AE(s).

7.1.9 Follow-Up Visits

Follow-Up visit week numbers for visits 1 through 6 are calculated from the number of weeks after Day 28 visit. During Follow-Up, participants will be followed at:

1. Follow-Up Visit 1 (Week 4)
2. Follow-Up Visit 2 (Week 8)
3. Follow-Up Visit 3 (Week 12)
4. Follow-Up Visit 4 (Week 16)
5. Follow-Up Visit 5 (Week 20)
6. Follow-Up Visit 6 (Week 24)

Follow-Up will include a scheduled telephone call, text message, or encrypted email message.

At Follow-Up Visit 1 (Week 4) and Follow-Up visit 6 (Week 24) the participant will also be asked to complete the WHOQOL survey in one of two ways:

a. Study team member will read the questions and record the participant's answers directly into REDCap or
b. The participant will complete the survey via an encrypted and personalized email link sent directly to the participant through REDCap

If the participant cannot be contacted directly, at least 3 attempts are to be made and documented in the research chart. If this occurs, the Follow-Up visit will consist of a review of the participant's medical record or documentation of communication with another physician treating the participant.

7.1.10 Follow-Up Assessments

The following information will be reviewed and/or collected during safety checks and Follow-Up visits:

1. Serious adverse events and AESIs
2. Medically attended AEs
3. Medical history
4. Concomitant Medications
5. Antibiotic use
6. Interim history of MDRO infection (and attempt to recover isolate from laboratory if available)
7. Weight (by standing scale when possible, otherwise bed scale will be used)
8. WHOQOL (week 4 and week 24)

7.2. Measures to Minimize Bias: Randomization and Blinding

This is an open label study of MT and participants and investigators will not be blinded to treatment. Effect size estimates will be performed after data lock.

7.3. Study Intervention Compliance

Study intervention administration compliance will be assessed by review of visit source documents. Compliance is expected to be high, as this investigational product for this study will be administered by a nurse or physician.

7.4. Concomitant Therapy

For this protocol, a prescription medication is defined as a medication that can be prescribed only by a properly authorized/licensed clinician. As eligible participants are admitted to long-term care facilities, all medications are expected to be prescribed and documented by the facility where the participant resides. Medications to be reported in the Case Report Form (CRF) are concomitant prescription medications, typically over-the-counter medications and supplements.

Concomitant medication logs will be completed during the screening visit and reviewed with participants or clinical staff at each visit. Antibiotics and some medication classes that are not conventional antibiotics (e.g., anti-psychotics) are known to influence microbiome composition and are important to consider in efficacy and exploratory analyses.

7.5. Rescue Medicine

For the purposes of this protocol, rescue medicines are most relevant in the context of infection after IP administration. The study site will not provide medications or pay costs for treatment for infection after IP administration.

8. Study Assessments and Procedures

8.1. Population and Procedures:

- The target enrollment for Sentinel REACT is 1-2 long-term care facilities (e.g. ventilator-capable skilled nursing facilities [vSNF] and long-term acute care hospitals [LTACH]) with an expected census of 20 to 60 patients per facility.
- After facility enrollment, under the APPS protocol (Emory IRB STUDY00004199) point-prevalence MDRO surveillance cultures of swabs collected from 3 body sites (axillae, inguinal fold, and peri-rectal swabs) from all assenting patients on participating ward(s). Swabs are processed in a central research (non-CLIA approved) laboratory in the Investigational Clinical Microbiology Core (ICMC) at Emory University using selective/differential media for isolation of extended-spectrum beta-lactamase producing Enterobacterales [ESBL], carbapenem-resistant Enterobacterales [CRE],

carbapenem-resistant *Pseudomonas aeruginosa* [CRPA], vancomycin-resistant *Enterococcus spp* [VRE], and *Clostridioides difficile*).

- Point-prevalence sampling will be repeated at four weeks (± 7 days) after first participant is treated with MT to estimate the effect of the microbiome therapy on MDRO prevalence and incidence rates at the ward and patient levels.
- All patients (or their legally-authorized representative for healthcare decisions) who are MDRO-positive during the first prevalence sampling time point will be approached for consent for microbiome therapy (via feeding tube when present or via enema when no feeding tube is present).

8.2. Efficacy Assessments

8.2.1. Procedures that will be completed as part of routine care

Participation in this study will not influence routine clinical care in general or diagnosis or treatment of infectious diseases in particular. Charts of MDRO-positive participants identified in the APPS study at the randomized time point will be reviewed to screen potential eligibility by demographic and clinical data. Specific demographic and clinical variables to be collected at screening include date of birth, age, race, ethnicity, medical history (including history of immunocompromised status, solid organ transplant, prior FMT or other microbiome therapeutic), surgical history, history of MDRO infection, reason for hospitalization, concomitant medications, length of stay while admitted, discharge location after hospitalization, location at 30-days after discharge (e.g. home, skilled nursing facility, rehabilitation facility, long-term acute care hospital, hospice), billing records for hospitalization, clinic visits, inpatient medications, imaging studies, and procedures. Collection of data for participants screened for eligibility is needed to evaluate for potential selection biases or non-random factors that may be associated with declining MT.

For the purposes of improving the design of future studies, to identify features associated with interest or declining FMT, and to compare clinical outcomes of those receiving FMT to eligible patients who do not. Frequency of infection (MDRO and non-MDRO) in the six months prior to eligibility and six months after eligibility will be compared in MT recipients vs contemporaneous controls who were admitted to the facility but did not receive MT. For the purposes of comparing MT-treated participants to contemporaneous controls for potential confounders, demographic and clinical characteristic data (age, sex, race, ethnicity, length of stay of present admission, total number of inpatient days, presence of tracheostomy, central line, urinary catheter, problem list, antibiotics prescribed, imaging study results, microbiology tests ordered, positive microbiology results) will be abstracted by data pull from Epic (with chart review validation on a subset of up to 50% of potential control patients) from patients admitted to the participating facility but not treated with MT.

For the purposes of assessing frequency of infection after MT compared to controls, any positive culture associated with documented symptoms, physical examination findings, or radiographic abnormalities suggestive of infection will be classified as an infection episode. If a new positive clinical culture isolates a bacterium that is of the same species and antibiotic susceptibility profile (with ≤2 discrepant antibiotic susceptibility results) of an isolate from the previous 12 months, such an isolate would be classified as a recurrence of a baseline infection. Clinical bacterial isolates that are identified by automated review of facility culture results or provider referral in search of potential participants will be collected and stored for future use.

A substantial fraction of MDRO-positive eligible patients admitted to participating facilities are expected to decline participation in the Sentinel REACT trial. Potentially eligible participants who decline enrollment will be asked to consider participation in **Group A** for collection of more frequent stool or skin swabs alone at the same time point or participation in **Group B** by providing consent for review of medical records alone.

8.3. Safety Assessments

8.3.1. Assessment of Adverse Events

AEs will be assessed by discussion with participants and medical chart review and documented at each visit. An equal effort will be made to obtain documentation and bacterial isolates from medically attended AEs that occurred at other facilities with participant release for these requests from other facilities.

Hard copies of the written visit worksheets will be provided for use as source document worksheets for recording data not derived from the medical record for each participant enrolled in the study. The medical record will be considered the source documentation for AEs that were documented in the medical record. Study staff/investigators may elect to record data directly in REDCap (the electronic case report form or eCRF). Data recorded in the electronic case report form (eCRF) derived from source documents should be consistent with the data recorded on the source documents and validated with periodic monitoring.

All Adverse Events (AEs) must be reported in routine study data submissions to the SI on the AE log forms. AEs reported through expedited processes (e.g., reported to the IRB, FDA, etc.) must also be reported in routine study data submissions. Solicited adverse events will be collected daily from participants during safety checks from Days 0 to 7.

Unsolicited AEs will be collected from participants for 28 days after each IP dose. Serious adverse events (SAEs) and adverse events of special interest (AESIs, see section 23.5.7) will be collected monthly from the first intervention until six months after the last dose, censored by death or loss to follow up. The study team will meet at least twice per year

to review the safety of the study with the DSMB, submit annual continuing review reports to the IRB, and annual reports to the FDA.

All SAEs and AEs that occur after intervention will be followed to adequate resolution or stabilization of the event(s) with relevant clinical assessments and laboratory tests as clinically appropriate as determined by the Sponsor-Investigator's satisfaction. AEs, actions taken because of AEs, and follow-up results will be recorded in the participant's medical record if not already documented.

9. Statistical Analysis Plan

9.1. General Approach

Measures of central tendency will be reported as median (Q1 to Q3) or mean (standard deviation) as appropriate for distribution of the reported value. Differences in central tendency will be tested by t-test or Mann-Whitney test as appropriate. Differences in proportion will be tested by chi-square or Fisher's exact test as appropriate. A p-value of <0.05 will be considered statistically significant. Exploratory analyses of high dimensional data (e.g. taxonomic comparisons, functional gene/metabolite comparisons) will be performed by Kruskal Wallis test, followed by pairwise tests as appropriate, with adjustment for multiple comparisons by the Benjamini-Hochberg method with a false-discovery rate of 0.05.

9.2. Planned Interim Analyses

Given the dynamic regulatory landscape of microbiota therapeutics in general and FMT in particular, the Sponsor-Investigator may elect to pause the study and conduct interim efficacy and futility analyses in the event any of the following developments:

- FDA places the study on clinical hold due to an issue that is not (in the opinion of the Sponsor-Investigator) expected to be resolved in > 18 months.
- Low facility or participant enrollment.

10. Data and/or Specimen Banking

10.1. Future Use of Stored Specimens and Data

Data collected for this study will be analyzed and stored at Emory University. After the study is completed, the de-identified, archived data will be stored at Emory, for use by other researchers including those outside of the study.

De-identified biological samples will be stored at Emory University. Cultured specimens (e.g. stool, skin swabs) and isolates will be stored in the Woodruff Memorial Research Building,

Health Sciences Research Building, or the Emory Investigational Clinical Microbiology Core facility. These samples could be used to research the causes of MDRO colonization, its complications and other conditions for which individuals with microbiome abnormalities are at increased risk, and to improve treatment. The repository at Emory University will also be provided with a code-link that will allow linking the biological specimens with the phenotypic data from each participant, maintaining the blinding of the identity of the participant.

At any time, an individual participant can choose to withdraw consent to have biological specimens stored for future research. When the study is completed, access to study data and/or samples will be provided through Emory University.

If a participant agrees to participate in this study, the participant will be assigned a study ID number and the Study Coordinators/data collectors will be instructed to collect the pertinent data/samples for this study.

11.Sharing of Results with Participants

MDRO analyses, which includes the following tests:

- **Skin swabs**: Participants will have their axillae and inguinal crease swabbed with culture swabs to test for MDROs. **Participants will not be informed of these results** as these assays will be performed in a research laboratory space that is not CLIA-certified and the results are not validated for reporting in the medical record.
- **Stool sample or swab culture**: Participants will have stool or perirectal/anal swab sampling. **Participants may be informed of these results in aggregate if they wish** (i.e. whether or not any swab was positive for any target MDRO but not the specific MDRO category) as these results do affect study participation eligibility.
- **Stool metagenomic sequencing**: Stool samples will be sent for metagenomic sequencing. **Participants will not be informed of these results,** as these results do not affect study participation eligibility.

Urine/serum pregnancy testing: Women of childbearing potential will have a pregnancy test performed. If the test is positive, their participation will be on hold until after they are no longer pregnant or breastfeeding. **Participants will be informed of these results**, as these results do affect study participation eligibility.

There are no direct benefits to the subject for extra specimens collected or from the secondary research. No results from secondary research will be entered into the subject's medical record.

Unless specified above, incidental findings will not be shared with the subject, including medically actionable incidental findings, unless required by law.

In the event that the researchers learn something new about risks of participating in this study, participants will be notified and can decide if they want to continue with study participation. Participants may be asked to sign a new consent form that includes the new risk information if they decide to stay in the study.

12. Study Timelines

A participant's study duration is anticipated to be 7-8 months and will consist of screening followed by up to one month for study visits and 6 months of safety follow-up, by monthly telephone visits with study staff. This study is anticipated to run for one years.

See **Appendix A** for **Table 6**: Schedule of Activities.

12.1. End of Study Definition

A participant is considered to have completed the study once all phases of the study are complete, including the last follow up visit or the last scheduled procedure. The end of the study is defined as completion of the last visit shown in the Schedule of Activities (SOA), as detailed in **Table 6** (see **Appendix A**).

13. Inclusion and Exclusion Criteria

13.1. Study Population

All genders, races, and ethnic groups (inclusive of women and minorities) will be recruited for this trial equally. Potential participants will be identified during MDRO prevalence sampling conducted under the APPS protocol.

During screening, patients will be approached by trained staff to discuss the study, review the informed consent form, and obtain informed consent for the Sentinel REACT and sub-studies. Whenever possible, the informed consent form will be provided in advance of the informed consent discussion. To enhance participant retention, study staff will remind participants of upcoming visits by text message or telephone call, and full compensation will be provided after last follow-up (though partial compensation will be provided for earlier study visits). Based on our prior clinical studies, we do not anticipate barriers to enrolling women or minority participants based on this recruitment and retention approach.

13.2. Human Subjects Research Review

Informed consent will be obtained from participants or their legally-authorized representative (LAR) after discussion of study aims, potential risks of IP and benefits reported in other settings with teach-back confirmation of participant understanding.

It is the responsibility of the investigator to submit this protocol, the ICF, relevant supporting information and all types of subject recruitment information to the IRB for review, and all must be approved prior to start of participant enrollment. Prior to implementing changes in the study, the IRB must approve any revisions of informed consent documents and amendments to the protocol unless there is an urgent subject safety issue.

13.3. Inclusion Criteria

A potential participant will only be considered eligible for the study if they meet all of the following criteria:

1. Be able to (or have available Legal Authorized Representative who is able to) understand and willing to sign a written informed consent document.
2. Be at least 18 years old at the time of consent.
3. Be able and willing to comply with all study protocol requirements, including willing to receive FMT through feeding tube or as retention enema.
4. Be colonized with a target MDRO (CRE, VRE, ESBL, MDR *Pseudomonas,* and/or *C. difficile)* as detected by bacterial culture of stool or peri-rectal swab.
5. Be willing to discontinue antibiotics, probiotics or other microbiota restoration therapies, and PPIs at least one day prior to study Day 0 upto Day 28.
6. The effects of the IP on the developing human fetus are unknown. For this reason, women of child-bearing potential (WOCBP) and men must agree to use adequate contraception (hormonal or barrier method of birth control; abstinence) prior to study entry and for the duration of study participation.
7. If potential participant is male and is sexually active with a partner of childbearing potential, the participant agrees to practice at least one effective method of birth control for the duration of the study.
8. If WOCBP, is participant willing to undergo urine human chorionic gonadotropin (hCG) testing on the day of FMT? Note that test result must be negative to proceed with FMT.

Potentially eligible participants may be unable to provide consent due to medical status (e.g. dementia, encephalopathy, endotracheal intubation, sedation). In these cases when participants are unable to provide consent, permission to enroll will be obtained from legally authorized representatives (LAR) in order of legal priority (e.g. durable power of attorney for health care, court-appointed guardian for health care decisions, spouse, adult child). This process will be detailed in a subsequent section of the protocol.

13.4. Exclusion Criteria

A subject will be excluded in the study if they meet any of the following criteria:

1. Be pregnant, breastfeeding, lactating, or planning a pregnancy during study duration (through 4 weeks after the last dose of investigational product, or IP), if WOCBP.
2. Have known uncontrolled intercurrent illness(es) such as, but not limited to:
    a. Symptomatic congestive heart failure
    b. Acute coronary syndrome
    c. Cardiac arrhythmia
    d. Untreated *in-situ* colorectal cancer
    e. Toxic megacolon
    f. Ileus
    g. Positive stool studies without completion of treatment course (including ova and parasites, *Salmonella spp, Shigella, Campylobacter*, and other enteropathogens).
3. Have any other intercurrent acute illness that in the opinion of the investigator will preclude subject from entering the study.
4. Be on systemic antibiotics for any reason other than treatment of recent MDRO infection or clear anticipated need for antibiotics during the follow up period that cannot be rescheduled (e.g. fluoroquinolone prophylaxis for percutaneous nephrostomy tube exchange). Participants must complete the planned antibiotic course by study Day -1.
5. Inability to discontinue proton-pump inhibitor therapy.
6. Have a compromised immune system, defined as:
    a. AIDS with CD4+ T-cell count <200 and any detectable HIV viral load.
    b. Absolute neutrophil count (ANC) <1,000 neutrophils / mL on day of enrollment.
    c. Active malignancy requiring intensive induction chemotherapy, radiotherapy, or biologic treatment within 2 months of enrollment.
    d. History of hematopoietic cell transplantation, either allogeneic or autologous in the last 1 year.
7. Have a history of significant food allergy that led to anaphylaxis or hospitalization.
8. Have a life expectancy of 24 weeks or less
9. Have any condition that, in the opinion of the investigator, might interfere with study objectives or limit compliance with study requirements, including but not limited to:
    a. Known active intravenous drug or alcohol abuse
    b. Psychiatric illness
    c. Social situation
10. Participated in an investigational study that also meets one of the following criteria:
    a. Received an interventional agent (drug, device, or procedure) in the last 28 days
    b. Enrollment in any other interventional study for MDROs.
11. Have a history of grade III or grade IV hemorrhoids or active, uncontrolled pain or

bleeding (will exclude from enema route of MT administration).

13.5. Lifestyle Considerations

During this study, participants are asked to refrain from receptive anal intercourse until the last biological specimen (stool sample) is collected.

14. Vulnerable Populations

Individuals who are vulnerable to coercion or undue influence (pregnant women, human fetuses, or neonates of uncertain viability or non-viable neonates, prisoners, and minors/children) are not eligible to participate in this study. When consent cannot be obtained from the participant due to neurological status, legally authorized representatives (LARs) will be approached to consider study participation and provide informed consent.

15. Local Number of Participants

In order to reach target enrollment of up to 20 MT-treated participants, we plan to screen up to 100 MDRO-positive potential participants from APPS for eligibility.

16. Recruitment Methods

16.1. Strategies for Recruitment and Retention

We to enroll all participants within one month from the randomized periodic point prevalence sampling performed under the APPS protocol. To retain participants, a fraction of compensation for time and travel will be provided after the last study visit. Based on our prior clinical studies, we do not anticipate barriers to enrolling women or minority participants based on this recruitment and retention approach.

17. Withdrawal of Participants

17.1. Discontinuation/Withdrawal of Study Intervention

Discontinuation of further IP (off-treatment) does not mean discontinuation from the study, and remaining study procedures such as sampling and monitoring for AEs should be completed as indicated by the study protocol. If a clinically significant finding is identified

(including, but not limited to changes from baseline) after enrollment, the investigator or qualified designee will determine if any change in level of participation is needed. Any new clinically relevant finding that is not included in the patient's baseline medical history will be reported as an adverse event (AE).

17.2. Participant Discontinuation/Withdrawal from the Study

Participants are free to withdraw from participation in the study at any time after notifying study staff. In addition, an investigator may discontinue any further IP administration or withdraw a participant from the study for the following reasons:

1) Pregnancy
2) Significant study intervention non-compliance or non-addressable feasibility challenges with IP administration (e.g. anatomic change, interim clinical need for bowel rest)
3) If any clinical adverse event (AE), laboratory abnormality, or other medical condition or situation occurs such that continued participation in the study would not be in the best interest of the participant
4) Disease progression which requires discontinuation of the study intervention
5) If the participant meets an exclusion criterion (either newly developed or not previously recognized) that precludes further study participation
6) Participant is unable to receive the assigned treatment for 30 days

The reason for participant discontinuation or withdrawal from the study will be recorded on the Discontinuation Case Report Form (CRF) and will also be documented in REDCap. Subjects who sign the informed consent form but do not receive the study intervention may be replaced. Subjects who sign the informed consent form, receive the study intervention and subsequently withdraw, or by PI discretion are withdrawn or discontinued from further IP administration during the study, may be replaced.

17.3. Lost to Follow-Up

A participant will be considered lost to follow-up if they are discharged from the participating facility and fail to return for two scheduled visits and are unable to be contacted by the study team. The following actions must be taken if a participant fails to return to the clinic for a required study visit:

1) The site will attempt to contact the participant and reschedule the missed visit within window and counsel the participant on the importance of maintaining the assigned visit schedule and ascertain if the participant wishes to and/or should continue in the study.
2) Before a participant is deemed lost to follow-up, the investigator or designee will make every effort to regain contact with the participant (where possible, 3 telephone calls, text messages, and, if necessary, a certified letter to the

participant's last known mailing address or local equivalent methods). These contact attempts should be documented in the participant's study file.

3) Should the participant continue to be unreachable, they will be considered to have withdrawn from the study with a primary reason of lost to follow-up.

17.4. Off-Study Criteria

A participant will be removed from the study when any of the following criteria apply:

1) Follow-up requirements have been completed
2) Lost to follow-up
3) Withdrawal of consent for participation
4) Inability to continue with study visits or treatment
5) Death or imminent death anticipated

The reason for taking a participant off study, and the date the participant was removed, will be documented in REDCap. The investigators have the right to stop a participant's participation in this study without their consent for any reason, including if it is believed to be the best interest of the participant, or if the participant were to object to any future changes that might be made in the study plan.

Should a participant choose to withdraw from the study, the investigators will not be able to withdraw the information that has already been used or shared with others to carry out related activities such as oversight, or that is needed to ensure quality of the study. To withdraw consent, participants will be instructed to do so in writing, by contacting the Sponsor-Investigator.

18. Risk to Participants

18.1. Known Potential Risks

The recognized risks related to MT are related to the instillation procedure and to the transplanted material itself. The use of MT rapidly increased after the landmark publication of a trial of duodenal infusion of fecal microbiota for the treatment of RCDI in 2013.[12] Since that time, it is thought that tens of thousands of treatments manufactured by the large, public stool bank OpenBiome, as well as by academic stool banks such as the one maintained at Emory, have been administered. A large prospective FMT registry has been created but is not considered a comprehensive surveillance program.[18] There is likely a publication bias against reporting incidences of MT treatment that are unsuccessful and in reporting potential adverse events that are directly attributable to the transplanted material.

There have been two published reports of documented transmission of pathogens that were not included in donor screening tests that led to infections.[19,20] in one case, the death

of an immunocompromised recipient who received a dose that was contaminated with an ESBL-producing *E. coli*.[19] These two episodes led to the FDA requiring enhanced screening of donors for MDROs and *E. coli* pathotypes for all MT studies conducted under an IND and to issue separate safety alerts about the risk of pathogen transmission after FMT that resulted in gastrointestinal infection:

- https://www.fda.gov/vaccines-blood-biologics/safety-availability-biologics/important-safety-alert-regarding-use-fecal-microbiota-transplantation-and-risk-serious-adverse
- https://www.fda.gov/vaccines-blood-biologics/safety-availability-biologics/safety-alert-regarding-use-fecal-microbiota-transplantation-and-risk-serious-adverse-events-likely)

MT has been studied in a number of prospective and retrospective studies, which did not report any similar AEs that were attributed to the transplanted material itself, which provides evidence that the frequency of these infection risks, though serious, is relatively low.[13–17]

In addition to bacterial pathogens, the GI tract is a recognized site of replication for the SARS-CoV-2, the virus that causes COVID-19. In early 2020, the FDA moved to put all MT studies on partial clinical hold until feces donor screening procedures to mitigate risk of SARS-CoV-2 transmission could be reviewed. This change was also accompanied by an FDA safety alert:

- https://www.fda.gov/vaccines-blood-biologics/safety-availability-biologics/safety-alert-regarding-use-fecal-microbiota-transplantation-and-additional-safety-protections

Expected AEs potentially related to recurrent CDI (from which most safety data for MT/FMT are available), MT, or the delivery procedure are summarized in the following list.

Table 1: Risks Associated with the Investigational/Study Agent (MT):

Very Common:	Common:	Rare:	Other:
Diarrhea	Nausea	Pyrexia (Fever ≥ 37.8° C (100.0°F))	Belching
Abdominal Pain	Constipation	Fatigue	Colitis
	Flatulence (gas)	Chills	Puncture of Intestine
	Abdominal Distension	Vomiting	Disease Transmission from the donor to recipient
	Urinary Tract Infection	Hypotension	
		Anorectal Irritation	
		Rectal bleeding	

Table 2: **Adverse events considered to be associated with the study procedure:**

These are the risks associated with the rectal retention enema procedure itself. Rare but serious (Less than 1% chance that this will happen):

Rare but Serious:
Bleeding
Perforation or tears
Severe abdominal pain
Cardiovascular events, such as a heart attack, low blood pressure, or the heart skipping beats or beating too slow
Death related to the procedure is an extremely rare event

Table 3: Risks Associated with Stool Samples

Uncommon:
Discomfort
Inconvenience

Table 4: Risks Associated with Perirectal Swabs

Occasional:	Uncommon:
Discomfort	Bleeding

Emory University has developed an intensive stool donor screening program, which includes comprehensive and ongoing stool and blood testing as well as frequent review of the donor's health history, current health status, and an assessment of risky lifestyle behaviors that predispose the donor to contracting transmissible disease, primarily human immunodeficiency virus (HIV), Hepatitis A, B, and C, syphilis, and MDROs.

The MT product that will be used in this study is manufactured utilizing donor and source material screening processes that were developed in conjunction with the FDA. These processes will be continuously monitored and updated as needed to ensure FDA compliance.

Therefore, the body of safety and efficacy evidence for MT and other human stool products, along with the rigor of Emory's donor screening process, and implementation of quality assurance testing at all stages of manufacturing supports a favorable benefit:risk profile for the use of MT in the reduction of MDRO colonization. The Sentinel REACT study is designed to collect additional safety data for MT in patients admitted to LTACHs and vSNFs as requested by the FDA.

18.2. Long-term IP risks

As the human microbiome is increasingly being recognized as potentially influencing many human physiologic systems, the long-term IP risks are incompletely understood. It has been nearly 10 years since publication of the landmark publication of FMT for RCDI in 2013 and the aforementioned infection alerts related to pathogen transmission are the only reports related to the transplanted microbiota itself. There is a theoretical long-term risk of colorectal cancers, inflammatory bowel disease, or immunologic disorders after microbiome therapy given the association data that have been published but no such cases have yet been recognized or reported.

18.3. Procedural risks

The procedural risks of MT are similar to risks associated with other suspensions administered via feeding tube (including aspiration, pneumonia or pneumonitis from aspiration, clogging or malfunctioning of tube requiring replacement, or dislodging of tube) or enema (discomfort with enema tube placement or volume of instillation, bleeding, bowel perforation or tear). The procedural risks associated with this study are detailed in Tables 1-4, in section 16.2. To minimize these potential risks, the MT will only be administered by trained staff and participants will be monitored during and closely after the procedure.

18.4. Reproductive Risks:

Participants will be advised that the study agent and intervention used in this research study may affect an egg, sperm, embryo, or fetus. Although these risks are currently unknown, participants who are of reproductive potential will be advised that they should not:

- Donate eggs or sperm
- Become pregnant in any way, including in-vitro fertilization (sometimes called "I-V-F")
- Nurse a baby

<u>Preventing Pregnancy</u>

The Screening section of the consent form is where participants will provide information about the status of their reproductive potential.

Participants who are of reproductive potential must agree to the use of contraceptives during heterosexual intercourse. Counseling about preventing pregnancy can be provided. During the consent process, participants will be told of the importance of not becoming pregnant while on study. Should pregnancy occur, participants will need to let the study doctor know. If a participant becomes pregnant while on study, they will not be administered any additional MT but will be asked to continue to participate in AE monitoring.

Participants who are not of reproductive potential will be asked to indicate how they know this (e.g., surgery, age, hormonal testing, or abstinence).

18.5. Non-Physical Risks:

Because of side effects or the time required for tests and clinic visits required for this study, a participant's schedule will be impacted.

In the event that the researchers learn something new about risks of participating in this study, participants will be notified and can decide if they want to continue with study

participation. If a participant decides to remain in the study, they may be asked to sign a new consent form that includes the new risk information.

Participants will be advised that there may be side effects from the study agent or procedures that are not known at this time. One risk that participants will be informed of, is that they may receive a study agent/intervention that does not help treat their condition or may make their condition worse. Another risk is that there may be side effects.

19. Potential Benefits to Participants

Based on the scientific literature and data generated at Emory, we expect that MT may safely reduce colonization with antibiotic resistant bacteria. We also expect that by offering FMT to all MDRO positive patients admitted to a long-term care facility that we there may be indirect benefits of reduced MDRO prevalence, infection incidence rates, and transmission.

19.1. Immediate potential IP benefits

The potential immediate MT benefits are expected to include reduction in MDRO intestinal colonization, and possibly reduction in recurrent infection with MDROs or non-MDRO pathogens. Participants may also potentially have improved quality of life and decreased healthcare cost/utilization or admission if they have fewer recurrent infections that require medical attention.

19.2. Long-range potential IP benefits

The long-range potential MT benefits are less understood but may be amenable to study in future retrospective studies.

20.Compensation to Participants

For each qualifying study visit/cycle, participants will be compensated as shown in the table below**.** If participants complete all study visits, participants may receive a maximum of $**200.00 USD**. For participants in control Group A, provided they complete 5 study visits, they will be compensated $50.

Table 5: Compensation for participation

**Qualifying Study Visit**	**Reimbursement (USD)**
Day 0 completed:	$50.00
Day 7 completed:	$25.00
Day 14 completed:	$25.00
Day 21 completed:	$25.00
Day 28 completed:	$25.00
Follow-up calls completed:	$50.00
**Total maximum compensation:**	**$200.00**

21.Data Management and Confidentiality

21.1. Confidentiality and Privacy

Participant confidentiality and privacy is strictly held in trust by the participating investigators, their staff, and the sponsor(s) and their interventions. This confidentiality is extended to cover testing of biological samples in addition to participant clinical information. Therefore, the study protocol, documentation, data, and all other information generated will be held in strict confidence. No information concerning the study or the data will be released to any unauthorized third party without prior written approval of the sponsor. All research activities will be conducted in as private a setting as possible.

The study monitor, other authorized representatives of the sponsor, representatives of the Institutional Review Board (IRB), or regulatory agencies may inspect all documents and records required to be maintained by the investigator, including but not limited to, medical records (office, clinic, or hospital) and GCRC biorepository records for the participants in this study. The clinical study staff will permit access to such records.

The study participant's contact information will be securely stored at Emory University for internal use during the study. At the end of the study, all records will continue to be kept in a secure location for as long a period as dictated by the reviewing IRB, Institutional policies, or sponsor requirements.

Study participant research data, which is for purposes of statistical analysis and scientific reporting, will be stored in a dual-factor authenticated, encrypted REDCap database with identifiers flagged to be excluded upon data export. Exported data for analysis will not include the participant's contact or identifying information. Rather, individual participants and their research data will be identified by a unique study identification number. The study data entry and study management systems used by Emory research staff will be secured and password protected. At the end of the study, all study databases will be de-identified and archived at Emory, for use by other researchers including those outside of the study.

For the purposes of date and location-resolved epidemiologic analyses of transmission events, analytic limited datasets may be prepared for collaborators under an approved data use agreement (DUA). Data are routinely collected in healthcare operations when a badged employee moves within detectable proximity of readers that are installed throughout Emory Healthcare facilities. These badge data are indexed by a unique ID and include provider type and name, as well as dates, and times of proximity detection. For the purposes of epidemiologic analyses, analytic datasets will be created that are either fully deidentified or only contain limited identifiers of date and time of badge proximity detection. The following limited identifiers may be included in these datasets that are not protected health information:

- Microbiology: Specimen collection date, specimen result date
- Provider badge location dataset: badge location, date, and time

With the participant's approval and as approved by local Institutional Review Boards (IRBs), de-identified biological samples may also be collected in the Georgia Clinical Research Clinic (GCRC) research unit HG of Emory University Hospital. Samples will be transported by study staff members and will be stored at Emory University with the same goal as the sharing of data. Access will be controlled, and samples will only be accessible by the study investigators.

Cultured specimens (e.g. stool, skin swabs) and isolates will be stored in the Woodruff Memorial Research Building or the Emory Investigational Clinical Microbiology Core facility. These samples could be used to research the causes of MDRO colonization, its complications and other conditions for which individuals with microbiome abnormalities are at increased risk, and to improve treatment.

The repository at Emory University will have a code-link that will allow linking of the biological specimens with the phenotypic data from each participant, maintaining the blinding of the identity of the participant.

If a participant agrees to participate in the main study but not the optional sub-studies, the participant will be assigned a study ID number and the Study Coordinators/data collectors will be instructed to collect data/samples only on those aspects of the study to which the participant has agreed to participate. This will help prevent unauthorized inclusion of the patient's data/samples in the database/repository. Participants will be told that they have the option of choosing whether or not to participate in the optional swabbing and chart review sub-studies. Samples will be collected, stored for up to 10 years, and used for future, exploratory analyses.

21.2. Certificate of Confidentiality

To further protect the privacy of study participants, a Certificate of Confidentiality has been automatically issued by the Centers for Disease Control & Prevention (CDC). This certificate protects identifiable research information from forced disclosure. It allows the investigator and others who have access to research records to refuse to disclose identifying information on research participation in any civil, criminal, administrative, legislative, or other proceeding, whether at the federal, state, or local level. By protecting researchers and institutions from being compelled to disclose information that would identify research participants, Certificates of Confidentiality help achieve the research objectives and promote participation in studies by helping assure confidentiality and privacy to participants.

22.Plans to Monitor the Data to Ensure Safety of Participants and Data Integrity

☒ **More than minimal risk**

Select one of the following		
	☐ Medium Complexity	
	☒ High Complexity Category A	
	☐ High Complexity Category B<br>*If choosing this category for a study under an IND or IDE because you believe the*	

	study intervention does not significantly impact morbidity or mortality, please provide your rationale:	

23. Monitoring Table 2

Please address the specific details below. Please do not alter the table and leave all template text to assist in a quick review. If deemed not applicable, please provide rationale.

**DSMP Requirement**	**How this Requirement is Met**	**Frequency**	**Responsible Party(ies)**
Real-time review of participant data during initial data collection.	To ensure that all pertinent data is accurately captured. Real-time review will help prevent items from being overlooked.	This review will take place every time new information is obtained. As enrollment continues, SI will decide if pacing of review needs to be adjusted.	SI, Sub-Investigators, and Study Coordinator will review for the first five patients. Study Coordinator to review for subsequent enrollees.
Site Monitoring at pre-determined intervals: The Principal Investigator has a responsibility to ensure that the study is following all aspects of the protocol.	External monitoring for Sentinel REACT will be performed by IND2Results, a CRO. IND2Results will draft an independent monitoring plan.	The IND2Results monitoring plan, at minimum, will include monitoring after the first participant is enrolled, every six months while participants are receiving intervention and annually while participants are in follow-up.	Site monitoring by external monitor(s) will be performed by IND2Results according to the monitoring plan.
100% review of	To ensure that the	Reviewed at a minimum of	SI, Sub-

regulatory files	regulatory file is current and complete for this study, and contains all of the necessary regulatory documents, from study start-up, to study completion.	first and close-out visits<br><br>Review can occur more frequently if there is increased activity (modifications/amendments, revised protocols, revised consent forms, annual renewal submissions, reportable events submissions, etc.).	Investigators, Study Coordinator, External Monitor(s) from IND2Results
100% review of consent forms	To ensure that participants are enrolled with the most current version of the consent form and that all consent forms are filled out completely.	Consent forms will be reviewed at the following timepoints:<br><br>- after the first participant is enrolled<br><br>- six months after the first participant is enrolled<br><br>- every 12 months thereafter<br><br>- at the end of the study	SI, Sub-Investigators, and Study Coordinator will review after each facility visit to obtain consent. External Monitor(s) from IND2Results will also review consent forms according to the monitoring plan.
Review of credentials, training records, the delegation of responsibility logs (if applicable)	To ensure that all study team members have completed the necessary onboarding/training requirements to begin working on this study and to ensure that study-specific responsibilities are captured for each team member.	Review will take place at a minimum of study initiation and at study close-out, however, review may occur if new team members are added to the study staff roster.	Study Coordinator
Comparison of case report forms (CRF) to source documentation for accuracy and completion	To ensure that source data/information has been captured and transcribed accurately to case report forms/REDCap.	Review will take place at a minimum of study initiation and at study close-out, however, review may occur if new versions of CRFs are released.	Study Coordinator, External Monitor(s) from IND2Results
Review of	To ensure that Adverse	Adverse Events will be	SI, Sub-

documentation of all adverse events	Events are captured and reported in a timely manner.	reviewed by study staff at the following timepoints:<br>- Individual adverse events will be reviewed in real-time<br>- An aggregate of adverse events will be reviewed each week<br>All study drop-outs and protocol deviations/violations will be reviewed each month<br>CRO study monitor(s) will review Adverse Events after each facility has completed follow up and at study close out.	Investigators, Study Coordinator, and CRO Study Monitor(s)
Monitoring of critical data points (eligibility, primary and secondary endpoints)	To ensure complete documentation of critical elements such as eligibility criteria being met, and objectives being achieved.	Monitoring is required at the following timepoints (but may be done more frequently):<br>- after the first participant is enrolled<br>- six months after the first participant is enrolled<br>- every 12 months thereafter<br>- at the end of the study	SI, Sub-Investigators, Study Coordinator, and CRO Study Monitor(s)
Laboratory review of processing and storage of specimens	To ensure processing and storage integrity for all collected samples.	Reviewed at first and close-out visits and at least biannually<br>SI will decide if pacing of review needs to be adjusted, based on speed of patient enrollment.	SI, Sub-Investigators, laboratory personnel

Assessment of laboratory specimens stored locally	To ensure processing and storage integrity for all collected samples.	Reviewed at first and close-out visits and at least biannually<br>SI will decide if pacing of review needs to be adjusted, based on speed of patient enrollment.	Site PI, Sub-Investigators, laboratory personnel
Test article accountability review		Reviewed at first and close-out visits and at least biannually	IDS, GCRC staff
Accountability logs, dispensing records, and other participant records	To ensure that all IP dispensing counts are in alignment with number of enrollees.	At least biannually<br>SI will decide if pacing of review needs to be adjusted. Counts will be performed Reviewed at first and close-out visits and at least biannually	SI, Sub-Investigators, and Pharmacist
For FDA regulated studies, the following requirements apply:	To ensure this study is being conducted in accordance within the scope of federal and institutional regulations.	Timing, frequency, and intensity of monitoring	Study team: SI, Sub-Investigators, Pharmacist, Study Coordinator, and laboratory personnel. SI will decide which team member(s) will be tasked with particular items.
Monitoring methods (may include centralized, on-site, and self-monitoring)	To ensure this study is being conducted in accordance within the scope of institutional and federal regulations.	Monitoring for this study will be an ongoing and continual process. Monitoring will be done on-site by site investigators and contracted	Study team: SI, Sub-Investigators, Pharmacist, Study Coordinator, laboratory personnel, and

		CRO monitor(s).	External Monitor(s) from IND2Results
*For international studies, you are required to engage a CRO that is working in the site country and/or to consult with Emory's legal counsel regarding compliance with the country's clinical research regulations.			

23.1. Conflict of Interest Policy

The independence of this study from any actual or perceived influence, such as by the pharmaceutical industry, is critical. Therefore, any actual conflict of interest of persons who have a role in the design, conduct, analysis, publication, or any aspect of this trial will be disclosed and managed. Furthermore, persons who have a perceived conflict of interest will be required to have such conflicts managed in a way that is appropriate to their participation in the design and conduct of this trial. Emory University has established policies and procedures for all study group members to disclose all conflicts of interest and the SI will establish a mechanism for the management of all reported dualities of interest.

23.2. Safety Oversight/ Data Safety and Monitoring Board (DSMB)

Safety oversight will be under the direction of a Data and Safety Monitoring Board (DSMB) composed of individuals with the appropriate expertise, including clinical trials, infectious diseases, and antimicrobial resistance. Members of the DSMB should be independent from the study conduct and free of conflict of interest, or measures should be in place to minimize perceived conflict of interest. The DSMB will meet at least semiannually, after enrollment of the first participant, to assess safety and efficacy data on each arm of the study. The DSMB will operate under the rules of an approved charter that will be written and reviewed at the organizational meeting of the DSMB. At this time, each data element that the DSMB needs to assess will be clearly defined.

Minimal information expected to be routinely provided to the committee will include: participant accrual; IP updates (related to relevant manufacturing, administration, safety data); all grade 2 or higher unexpected adverse events that have been reported; summary of all deaths (occurring within 30 days of intervention, while being treated, and during active follow-up); efficacy data; audit results, and narrative summaries when anticipated to be helpful. Other information (e.g. culture results, laboratory values) will be provided upon request.

Within one week after meeting, the DSMB will provide its input to the SI in the form of a written, signed report. Information that raises any questions about participant safety will be addressed with the Overall Sponsor Investigator and study team.

The DSMB charter is attached as **Appendix B**.

23.3. Quality Assurance and Quality Control

The SI will oversee internal quality management of study conduct, data and biological specimen collection, documentation and completion. Quality control (QC) procedures will be implemented beginning with the data entry system and data QC checks that will be run on the database will be generated. Any missing data or data anomalies will be communicated for clarification/resolution.

Following written Standard Operating Procedures (SOPs), the monitor(s) will verify that the clinical trial is conducted and data are generated and biological specimens are collected, documented (recorded), and reported in compliance with the protocol, International Conference on Harmonisation Good Clinical Practice (ICH GCP), and applicable regulatory requirements (e.g., Good Laboratory Practices (GLP), Good Manufacturing Practices (GMP)).

23.4. Data Handling and Record Keeping

22.4.1 Data Collection and Management Responsibilities

Data collection is the responsibility of the clinical trial staff under the supervision of the SI. The investigator is responsible for ensuring the accuracy, completeness, legibility, and timeliness of the data reported. All written source documents will be completed in a neat, legible manner to ensure accurate interpretation of data.

Hard copies of written visit worksheets will be provided for use as source document worksheets for recording data for each participant enrolled in the study. Study staff/investigators may elect to record data directly in the REDCap electronic case report form (eCRF) provided the project is hosted on the part 11 compliant REDCap instance at Emory. Data recorded in the electronic case report form (eCRF) derived from source documents should be consistent with the data recorded on the source documents.

Clinical data (including adverse events (AEs), concomitant medications, and expected adverse reactions data) and clinical laboratory data will be entered into REDCap. Emory has a 21 CFR Part 11-compliant instance. The data system includes password protection and internal quality checks, such as automatic range checks, to identify data that appear inconsistent, incomplete, or inaccurate. Clinical data will be entered directly from the source documents (i.e. the electronic health record).

22.4.2 Study Records Retention

Study documents should be retained for a minimum of 2 years after the last approval of a marketing application in an International Conference on Harmonisation (ICH) region and until there are no pending or contemplated marketing applications in an ICH region or until at least 2 years have elapsed since the formal discontinuation of clinical development of the study intervention. These documents should be retained for a longer period, however, if required by local regulations. No records will be destroyed without the written consent of the sponsor, if applicable. It is the responsibility of the sponsor to inform the investigator when these documents no longer need to be retained.

22.4.3 Protocol Deviations

A protocol deviation is any noncompliance with the clinical trial protocol, International Conference on Harmonisation Good Clinical Practice (ICH GCP), or Manual of Procedures (MOP) requirements. The noncompliance may be either on the part of the participant, the investigator, or the study site staff. As a result of deviations, corrective actions are to be developed by the site and implemented promptly.

These practices are consistent with ICH GCP:

- 4.5 Compliance with Protocol, sections 4.5.1, 4.5.2, and 4.5.3
- 5.1 Quality Assurance and Quality Control, section 5.1.1
- 5.20 Noncompliance, sections 5.20.1, and 5.20.2.

It is the responsibility of the site investigator to use continuous vigilance to identify and report deviations within 5 working days of identification of the protocol deviation, or within 2 working days of the scheduled protocol-required activity. All deviations must be addressed in study source documents and reported to the SI. Protocol deviations must be sent to the reviewing Institutional Review Board (IRB) per their policies. The SI is responsible for knowing and adhering to the reviewing IRB requirements. Further details about the handling of protocol deviations will be included in the MOP.

22.4.4 Publication and Data Sharing Policy

The primary endpoint analysis results should be made public within 12 months after the Study Completion Date. The Study Completion Date is the date that the final participant was examined for the purposes of the primary outcome, whether the trial concludes per the prespecified protocol or was terminated at an earlier date. A full report of the non-primary endpoint outcomes should be made public no later than three years after the end of the study.

23.4.1. Clinical Monitoring

This is a greater than minimal risk study. Clinical site monitoring will be conducted to ensure that the rights and well-being of trial participants are protected, that the reported trial data are accurate, complete, and verifiable, and that the conduct of the trial is in compliance with the currently approved protocol/amendment(s), with International Conference on Harmonisation Good Clinical Practice (ICH GCP), and with applicable regulatory requirement(s).

- Monitoring for this study will be performed by an external monitor from the CRO IND2Results.
- Monitoring will be performed remotely for most documents but on-site when electronic documents are not available or not precluded by COVID-19 related restrictions.
- Monitoring will take place after the first participant, at six months, and then every twelve months while the study is ongoing, with a final monitoring review at end of study.
- The extent of monitoring will be 100% review of consent and eligibility criteria screens, and 100% review of safety (AE) and efficacy (MDRO culture & infection frequency) data.
- The Sponsor Investigator will be provided copies of monitoring reports within 14 days of study monitor visit.
- There are no international sites for this study.

23.5. Adverse Events and Serious Adverse Events

23.5.1. Definition of Adverse Events (AE)

An adverse event is any untoward medical occurrence associated with the use of an intervention in humans, whether or not considered intervention-related (21 CFR 312.32 (a)).

23.5.2. Definition of Serious Adverse Events (SAE)

An adverse event (AE) or suspected adverse reaction is considered "serious" if, in the view of either the investigator or sponsor, it results in any of the following outcomes: death, a life-threatening adverse event, inpatient hospitalization or prolongation of existing hospitalization, a persistent or significant incapacity or substantial disruption of the ability to conduct normal life functions, or a congenital anomaly/birth defect. Important medical events that may not result in death, be life-threatening, or require hospitalization may be considered serious when, based upon appropriate medical judgment, they may jeopardize the participant and may require medical or surgical intervention to prevent one of the outcomes listed in this definition. Examples of such medical events include allergic bronchospasm requiring intensive treatment in an

emergency room or at home, blood dyscrasias or convulsions that do not result in inpatient hospitalization, or the development of drug dependency or drug abuse.

Of note, hospitalization and intravenous (IV) antibiotic treatment of MDRO infections are anticipated to be a baseline inclusion criterion for all participants.

23.5.3. Classification of an Adverse Event

22.5.3.1 Severity of Event

Adverse Events (AEs) are to be graded by severity and causality. For adverse events (AEs) not included in the protocol defined grading system, the following criteria will be used to classify severity.

- **Mild** – Events require minimal or no treatment and do not interfere with the participant's daily activities.
- **Moderate** – Events result in a low level of inconvenience or concern with the therapeutic measures. Moderate events may cause some interference with functioning.
- **Severe** – Events interrupt a participant's usual daily activity and may require systemic drug therapy or other treatment. Severe events are usually potentially life-threatening or incapacitating. Of note, the term "severe" does not necessarily equate to "serious".

22.5.3.2 Relationship to Study Intervention

All adverse events (AEs) must have their relationship to study intervention assessed by the clinician who examines and evaluates the participant based on temporal relationship and clinical judgment. The degree of certainty about causality will be graded using the categories below. In a clinical trial, the study product must always be suspect.

- **Definitely Related** – There is clear evidence to suggest a causal relationship, and other possible contributing factors can be ruled out. The clinical event, including an abnormal laboratory test result, occurs in a plausible time relationship to study intervention administration and cannot be explained by concurrent disease or other drugs or chemicals. The response to withdrawal of the study intervention (dechallenge) should be clinically plausible. The event must be pharmacologically or phenomenologically definitive, with use of a satisfactory rechallenge procedure if necessary.
- **Probably Related** – There is evidence to suggest a causal relationship, and the influence of other factors is unlikely. The clinical event, including an abnormal laboratory test result, occurs within a reasonable time after administration of the study intervention, is unlikely to be attributed to

concurrent disease or other drugs or chemicals, and follows a clinically reasonable response on withdrawal (dechallenge). Rechallenge information is not required to fulfill this definition.

- **Potentially Related** – There is some evidence to suggest a causal relationship (e.g., the event occurred within a reasonable time after administration of the trial medication). However, other factors may have contributed to the event (e.g., the participant's clinical condition, other concomitant events). Although an AE may rate only as "possibly related" soon after discovery, it can be flagged as requiring more information and later be upgraded to "probably related" or "definitely related", as appropriate.
- **Unlikely to be related** – A clinical event, including an abnormal laboratory test result, whose temporal relationship to study intervention administration makes a causal relationship improbable (e.g., the event did not occur within a reasonable time after administration of the study intervention) and in which other drugs or chemicals or underlying disease provides plausible explanations (e.g., the participant's clinical condition, other concomitant treatments).
- **Not Related** – The AE is completely independent of study intervention administration, and/or evidence exists that the event is definitely related to another etiology. There must be an alternative, definitive etiology documented by the clinician.

22.5.3.3 Expectedness

Trained investigators will be responsible for determining whether an adverse event (AE) is expected or unexpected. An AE will be considered unexpected if the nature, severity, or frequency of the event is not consistent with the risk information previously associated in the literature with FMT.

23.5.4. Time Period and Frequency for Event Assessment and Follow-Up

The occurrence of an adverse event (AE) or serious adverse event (SAE) may come to the attention of study personnel during study visits and interviews of a study participant presenting for medical care, or upon review by a study monitor.

All AEs including local and systemic reactions not meeting the criteria for SAEs will be captured on the appropriate case report form (CRF). Information to be collected includes event description, date of onset, clinician's assessment of severity, relationship to study product (assessed only by those with the training and authority to make a diagnosis), and date of resolution/stabilization of the event. All AEs occurring while on study must be documented appropriately regardless of relationship. All AEs will be followed to adequate resolution.

Any medical condition that is present at the time that the participant is screened will be considered as baseline and not reported as an AE. However, if the study participant's condition deteriorates at any time during the study, it will be recorded as an AE.

Changes in the severity of an AE will be documented to allow an assessment of the duration of the event at each level of severity to be performed. AEs characterized as intermittent require documentation of onset and duration of each episode.Trained study staff will review and record all reported events and pre-specified solicited AEs on Day 0, 1, 2, 3, 4, 5, 6, Day 7, Day 14, Day 21, Day 28. All AEs will be monitored from Day 0 through the end of the study including in the follow-up 6 month period.

Trained study staff will record all reportable events with start dates occurring any time after informed consent is obtained until 7 (for non-serious AEs) or 30 days (for SAEs) after the last day of study participation. At each study visit, the study staff will inquire about the occurrence of AE/SAEs since the last visit. Events will be followed for outcome information until resolution or stabilization.

23.5.5. Adverse Event Reporting

All AEs must be reported in routine study data submissions to the SI on case report forms. AEs reported through expedited processes (e.g., reported to the IRB, FDA, etc.) must also be reported in routine study data submissions. Solicited adverse events will be assessed from participants on Days 0, 7, 14, and 28 and during the Follow-Up period. SAEs will be collected from the date of the first intervention through the end of study. AEs (including non-SAEs) will be collected by chart review, telephone call, text message, or electronic communication for 24 weeks from last visit of last cycle. The study team will meet approximately every six months to review the safety of the study with the DSMB.

Each participant will be monitored for the occurrence of AEs, including SAEs, beginning immediately after FMTadministration and/or swabbing. All SAEs and AEs will be followed to adequate resolution or stabilization of the event(s) with relevant clinical assessments and laboratory tests as clinically appropriate as determined by the Sponsor-Investigator's satisfaction. Related AEs, actions taken because of AEs, and follow-up results will be recorded in the participant's medical record.

Participants will be asked at each follow up time-point regarding presence or absence of the following solicited adverse events including abdominal pain, fever, diarrhea, constipation, flatulence, bloating, vomiting, and well-being qualitative survey instruments (i.e. WHOQOL) plus physical examinations per the schedule of assessments by the treating investigator for evidence of AEs. The questioning of participants regarding the possible occurrence of adverse events will also be generalized such as, "How have you been feeling since your last visit?" in addition to solicited AE review.

23.5.6. Serious Adverse Event Reporting

The study clinician will immediately report to the sponsor any serious adverse event, whether or not considered study intervention related, including those listed in the protocol or investigator brochure and must include an assessment of whether there is a reasonable possibility that the study intervention caused the event. Study endpoints that are serious adverse events (e.g., all-cause mortality) must be reported in accordance with the protocol unless there is evidence suggesting a causal relationship between the study intervention and the event (e.g., death from anaphylaxis). In that case, the investigator must immediately report the event to the sponsor.

All serious adverse events (SAEs) will be followed until satisfactory resolution or until the site investigator deems the event to be chronic or the participant is stable. Other supporting documentation of the event may be requested by the Data Coordinating Center (DCC)/study sponsor and should be provided as soon as possible.

The study sponsor will be responsible for notifying the Food and Drug Administration (FDA) of any unexpected fatal or life-threatening suspected adverse reaction as soon as possible, but in no case later than 7 calendar days after the sponsor's initial receipt of the information. In addition, the sponsor must notify FDA and all participating investigators in an Investigational New Drug (IND) safety report of potential serious risks, from clinical trials or any other source, as soon as possible, but in no case later than 15 calendar days after the sponsor determines that the information qualifies for reporting.

23.5.7. Events of Special Interest

Events of special interest that will prompt reporting to the FDA and IRB include:

- Suspected transmission of a pathogen from the IP to trial participant(s) (e.g. bacteria of same species and antibiotic susceptibility profile in more than one participant).
- If more than 2 participants experience the same Grade 3 Adverse Event (excluding admission for recurrence of MDRO infection or other baseline medical condition)
- New diagnoses of inflammatory bowel disease (IBD) in ≥2 treated participants.
- Gram negative or anaerobe blood stream infection within 14 days after IP without any alternate etiology.

23.5.8. Reporting of Pregnancy

If participants become pregnant during the course of the study, they will receive no further study interventions but will complete the remaining visits for their last cycle. Permission will be requested to follow pregnant women to their pregnancy outcome if longer than the planned study visits.

23.6. Unanticipated Problems

23.6.1. Definition of Unanticipated Problems (UP)

The Office for Human Research Protections (OHRP) considers unanticipated problems involving risks to participants or others to include, in general, any incident, experience, or outcome that meets all of the following criteria:

- Unexpected in terms of nature, severity, or frequency given (a) the research procedures that are described in the protocol-related documents, such as the Institutional Review Board (IRB)-approved research protocol and informed consent document; and (b) the characteristics of the participant population being studied;
- Related or possibly related to participation in the research ("possibly related" means there is a reasonable possibility that the incident, experience, or outcome may have been caused by the procedures involved in the research); and
- Suggests that the research places participants or others at a greater risk of harm (including physical, psychological, economic, or social harm) than was previously known or recognized.

23.6.2. Unanticipated Problem Reporting

FDA/Center for Biologics Evaluation and Research (CBER) will be notified by phone or fax within 48 hours if the study is halted for review.

The SI will be responsible for all communications with the FDA. The SI will report to the FDA, regardless of the site of occurrence, any serious adverse event after IP administration that meets the FDA's criteria for expedited reporting following the reporting requirements and timelines set by the FDA. The IND sponsor will also assess whether the event constitutes an unanticipated problem (UP) posing risks to subjects or others. This assessment will be provided to the Emory University IRB, which, in turn will make a final determination. If the Emory IRB determines an event is a UP it will notify the appropriate regulatory agencies and institutional officials.

Participating investigators will report to their local Risk Management office any participant safety reports or sentinel events that require reporting per institutional policy, such as:

- Electronic reporting
- Calling the EUH Risk Manager
- Calling a physician leader (e.g., Chief Quality Officer or Chief Medical Officer)

23.6.3. Reporting Unanticipated Problems to Participants

Participants will generally not be informed of unanticipated problems unless the potential benefits are expected to outweigh the potential risks of notification. For example, if an IP dose was found to be contaminated by a pathogen and multiple participants were administered IP from the same donor, the potential benefits to participants receiving IP for proceeding with diagnostics and treatment of infection could outweigh the potential risks of anxiety about receiving a contaminated dose.

23.7. Sponsor-Investigator Requirements

The Sponsor-Investigator is responsible for:

- **Instructing participants to report AEs**
- **Evaluating participant safety** including (S)AE assessment for seriousness, severity, and causality
- **Informing the IRB of SAEs as required per IRB guidelines**
- **Reporting of Non-serious AEs**: All AEs, regardless of seriousness, severity, or causal relationship to the IP, will be recorded in the participant's medical record if not already documented.
- **Reporting of IP exposure during Pregnancy**: If a participant or participant's partner becomes pregnant during 4-week period post-intervention, the treating investigator is required to request to continue follow up until the pregnancy has completed.
- **Reporting of Safety Observations by the Physician**: Any occurrence of the following events or outcomes a participant treated under this IND must be reported expeditiously by the Sponsor-Investigator to the FDA.
    - SAE within 24 hours after IP administration
    - Death of participant after IP exposure

23.8. Expedited Reporting

Investigators must report to the SI any serious adverse event (SAE) that occurs within 4 weeks of the last IP administration on the SAE form or other means approved by SI with IRB approval.

23.9. Halting Rules

Specific safety findings will result in temporary suspension of enrollment and treatment of enrolled participants until a safety review is convened, the objective of which is a decision as to whether the study should continue per protocol, proceed with caution, be further investigated, be discontinued, or be modified and then proceed. Suspension of enrollment (for a particular group or for the entire study) is another potential outcome of a safety review.

Subsequent review of serious, unexpected, and related AEs by the DSMB, IRB, the SI, or the FDA or relevant local regulatory authorities may also result in suspension of further trial interventions/administration of study intervention at a site. The FDA and SI retain the authority to suspend additional enrollment and study interventions/administration of study intervention for the entire study, as applicable.

23.10. Safety review triggers

Findings that will trigger a safety review are:

- Death
- One or more suspected as well as confirmed transmission of an infection from the IP to a study participant(s) (e.g. two or more participants with symptoms of new infection and positive culture with similar antibiotic susceptibility profile, average nucleotide identity, and or detection of antimicrobial resistance/virulence genes)
- If more than two participants experience the same Grade 3 Adverse Event classified as possibly related (excluding readmission for recurrence of known MDRO infection or other baseline medical condition)
- If one or more participants experiences a serious, unexpected adverse event
- One or more new diagnoses of inflammatory bowel disease (IBD) in IP-treated participants.
- One or more participants with Gram negative or anaerobe blood stream infection within 14 days after IP, and without any alternate etiology

In the event of a study pause, the SI will be responsible for halting enrollment and treatment and initiating a safety review within 48 hours. Results of this review will be presented to the DSMB.

For individual participants enrolled in the study: any participant who has an SAE related to study intervention or procedures will be ineligible for a second study intervention during the course of the study.

24. Provisions to Protect the Privacy Interest of Participants

Participant confidentiality and privacy is strictly held in trust by the study team and extends to cover testing of biological samples, and clinical information relating to participants.

The study protocol, documentation, data, and all other information generated will be held in strict confidence. No information concerning the study or the data will be released to any unauthorized third party without prior written approval of the sponsor.

All research activities will be conducted in as private a setting as possible. Participants will only interact with members of the study team for this study. The study participant's contact information will be securely stored at Emory University for internal use during the study. At the end of the study, all records will continue to be kept in a secure location for as long a period as dictated by the reviewing IRB, Institutional policies, or sponsor requirements.

Individual participants and their research data will be identified by a unique study identification number. The study data entry and study management systems used by Emory research staff will be secured and password protected. At the end of the study, all study databases will be de-identified and archived at Emory.

To further protect the privacy of study participants, a Certificate of Confidentiality has been automatically issued by the Centers for Disease Control & Prevention (CDC). By protecting researchers and institutions from being compelled to disclose information that would identify research participants, Certificates of Confidentiality help achieve the research objectives and promote participation in studies by helping assure confidentiality and privacy to participants.

Care will be taken to ensure that study-related questions, examinations, and procedures will not be intrusive.

25.Economic Burden to Participants

There are no costs, research or standard of care related, associated with the study. The sponsor will not pay for a participant's regular medical care. For participants at Emory with insurance, Emory will submit claims to the participant's insurance provider for items and services that are part of a participant's regular medical care. All other research activities will be paid for by the study sponsor.

There will be no other costs for participating in this study, other than basic expenses like transportation. If transportation costs or logistics are a barrier to someone participating in this study, the team will help arrange transportation for the participant. Participants will not be charged for any research activities.

26.Informed Consent

26.1. Consent/Assent and Other Informational Documents Provided to Participants

This study is open to adults, age 18 and older. Children/minors will not be enrolled in this study. We will not request a waiver or alteration of consent for this study. A physician on

the study team (either the SI or another treating investigator) will obtain informed consent from the participant or legally-authorized representative (LAR) by reviewing the study plan, benefits and risks, and alternative options. A Study Coordinator may obtain the administrative signatures on the informed consent form (ICF), but a physician on the study team must verbally obtain informed consent from the participant and document the process. The most recent version of the protocol and eligibility checklist will be used during the screening and enrollment process. All research activities, including the informed consent process, will be conducted in as private a setting as possible.

When reviewing the consent form with a potential participant, each participant will be encouraged to ask any questions that they might have. To ensure participants' understanding, participants will also be asked to "repeat back" elements of the study, so that a physician on the study team can assess whether or not the patient has an understanding of what will be involved, should they decide to go on study.

Once it has been determined that a patient is eligible for study inclusion, they will be approached for consent. Participants will be provided a link to an informational video that describes practical aspects of MT, donor screening, the procedure itself, as well as expected risks, benefits and alternatives to participation. Patients and their Legally Authorized Representative will be given ample time to review the consent form and to ask any questions that they might have.

As much time as needed will be devoted to the informed consent discussion. While this might vary from participant to participant, all participants will be given time to decide whether or not they want to enroll in the study. Participants will also be encouraged to discuss study participation with others, and participants will also be encouraged to ask questions.

To ensure ongoing consent after a participant enrolls in the study, participants will be asked if they are willing to continue in the study. This will be done on an ongoing basis. Participants will also be encouraged to ask any questions that they might have, as they progress throughout the study.

It is not anticipated that the amount of compensation being provided for participation would lead to coercion or undue influence. Participants will have ample time to review the consent form and will have the opportunity to discuss the study with family members. During the consent process, participants will be encouraged to ask questions and will have their questions answered.

26.2. Registration Process

After obtaining informed consent, participants will be registered with the Office of Clinical Research (OCR) in Emory OnCore. A copy of the informed consent form signature page and participant information will be sent within 24 hours of signature.

26.3. Registration Procedure Description

Eligible participants at Emory will be registered in OnCore, with the Emory Office for Clinical Research (OCR), and in an electronic data capture (EDC) system (e.g. REDCap). Registration must occur prior to study enrollment. Any participant not registered before will be considered ineligible and registration will be denied. A member of the study team will complete the protocol-specific eligibility checklist and an investigator will confirm eligibility.

Following registration, all eligible and willing participants may proceed to study activity. Issues that would cause delays should be discussed with the Sponsor-Investigator (SI). If a participant does not receive the assigned intervention following registration, the participant's registration will be updated as a screen failure, unless rescreening can occur within the previously specified 14-day window.

26.4. Non-English-Speaking Participants

Fluency in English is not an inclusion criterion for participation in this trial. If participants who do not speak fluent English wish to enroll, oral and written information will be provided in English in advance and verbally reviewed in its entirety with a fluent interpreter with the Emory short-form consent in their primary language. Emory's short form consents cover a wide range of languages, as does the interpretation service that Emory uses.

26.5. Adults Unable to Consent

Potentially eligible participants may be unable to provide consent due to medical status (e.g. dementia, encephalopathy, intubation, sedation). In these cases, permission to enroll will be obtained from legally authorized representatives (LAR) in order of legal priority (e.g. durable power of attorney for health care, court-appointed guardian for health care decisions, spouse, adult child).

27.Setting

This research study and all study and procedures will be conducted at Emory's Long-Term Acute Care facility, in Decatur, Georgia. Participants will be identified from patients with a positive screening culture performed during MDRO prevalence sampling with the APPS study and from clinician referral.

28.Resources Available

All necessary resources are available to safely undertake this research study, while maintaining compliance with all applicable regulations and IRB requirements are in place. Staffing, dedicated space to care for/treat patients for study visits and provisions for securing research documents (paper-based records created by the research team and electronic data) are all in place. The time planned to complete exploratory analyses for this study is five years.

Emory participants will be recruited from the Emory Long-Term Acute Care facility in Decatur, Georgia.

All study personnel assisting with this study will have undergone protocol training, including training on research procedures and their related duties and functions. Adverse Events will be assessed only by those with the training and authority to make a diagnosis. Staff who are responsible for the conduct, management, or oversight of study have completed Human Subjects Protection and ICH GCP Training.

29. References

1. CDC. Antibiotic Resistance Threats in the United States, 2019. Atlanta, GA; 2019. doi:CS239559-B
2. Chen L, Todd R, Kiehlbauch J, Walters M, Kallen A. Notes from the Field: Pan-Resistant New Delhi Metallo-Beta-Lactamase-Producing Klebsiella pneumoniae - Washoe County, Nevada, 2016. MMWR Morb Mortal Wkly Rep. 2017;66(1):33. doi:10.15585/mmwr.mm6601a7
3. Woodworth KR, Walters MS, Weiner LM, et al. Vital Signs : Containment of Novel Multidrug-Resistant Organisms and Resistance Mechanisms — United States, 2006–2017. MMWR Morb Mortal Wkly Rep. 2018;67(13):396-401. doi:10.15585/mmwr.mm6713e1
4. Woodworth MH, Hayden MK, Young VB, Kwon JH. The Role of Fecal Microbiota Transplantation in Reducing Intestinal Colonization With Antibiotic-Resistant Organisms: The Current Landscape and Future Directions. Open forum Infect Dis. 2019;6(7):1-9. doi:10.1093/ofid/ofz288
5. Saha S, Tariq R, Tosh PK, Pardi DS, Khanna S. Fecal Microbiota Transplantation for Eradicating Carriage of Multidrug-Resistant Organisms: A Systematic Review. Clin Microbiol Infect. April 2019. doi:10.1016/j.cmi.2019.04.006
6. Feldgarden M, Brover V, Haft DH, et al. Validating the AMRFinder Tool and Resistance Gene Database by Using Antimicrobial Resistance Genotype-Phenotype Correlations in a Collection of Isolates. Antimicrob Agents Chemother. 2019;63(11):1-19. doi:10.1128/AAC.00483-19
7. Lau MSY, Grenfell BT, Worby CJ, Gibson GJ. Model diagnostics and refinement for phylodynamic models. Rasmussen DA, ed. PLOS Comput Biol. 2019;15(4):e1006955. doi:10.1371/journal.pcbi.1006955
8. Prem K, Lau MSY, Tam CC, Ho MZJ, Ng L-C, Cook AR. Inferring who-infected-whom-where in the 2016 Zika outbreak in Singapore—a spatio-temporal model. J R Soc Interface. 2019;16(155):20180604. doi:10.1098/rsif.2018.0604
9. Lau MSY, Gibson GJ, Adrakey H, et al. A mechanistic spatio-temporal framework for modelling individual-to-individual transmission—With an application to the 2014-2015 West Africa Ebola outbreak. Ferrari M (Matt), ed. PLOS Comput Biol. 2017;13(10):e1005798. doi:10.1371/journal.pcbi.1005798

Appendix A: Schedule of Activities (SOA)

Table 6: Participant Schedule of Activities

**Schedule of Events for Study Participants in FMT Group**							
	**Day 0**	**Day 1, 2, 3, 4, 5, 6**	**Day 7**	**Day 14**	**Day 21**	**Day 28**	**Monthly Follow-up x 6**
**Stool sample or rectal swab**	✓		✓	✓	✓	✓	
**Physical exam**	✓						
**Targeted Symptom Review**	✓	✓	✓	✓	✓	✓	
**Medical History Review**	✓	✓	✓	✓	✓	✓	✓
**Medication Review**	✓	✓	✓	✓	✓	✓	✓
**Environmental (room) swabbing**	✓		✓	✓	✓	✓	
**Urine pregnancy test**	✓						
**Microbiome Therapy (MT)**	✓						
**Quality of Life Survey**	✓					✓	✓
**Phone call, text message, email or bedside visit**							✓

**Schedule of Events for Study Participants in Group A**						
	**Day 0**	**Day 7**	**Day 14**	**Day 21**	**Day 28**	**Monthly Follow-up x 6**
**Stool sample or rectal swab**	✓	✓	✓	✓	✓	
**Medical History Review**	✓	✓	✓	✓	✓	✓
**Medication Review**	✓	✓	✓	✓	✓	✓

**Environmental (room) swabbing**	✓	✓	✓	✓	✓	
**Quality of Life Survey**	✓				✓	✓
**Phone call, text message, email or bedside visit**						✓

**Schedule of Events for Study Participants in Group B**			
	**Day 0**	**Day 28**	**Monthly Review x 6 months**
**Medical History Review**	✓	✓	✓
**Medication Review**	✓	✓	✓

Table 6: Consolidation of Objectives, Endpoints and Endpoint Justifications

OBJECTIVES	ENDPOINTS	ENDPOINT JUSTIFICATION
**PRIMARY**		
1. Safety of the IP in patients admitted to long-term care facilities	1. IP safety will be measured as frequency and severity of AEs between Day 0 and Day 7 and reviewed by DSMB for consideration of proceeding to enrollment of additional sites.<br>2. IP safety will also be assessed as frequency and severity of AEs at day 30 and six months after last visit censored by last telephone visits or death, whichever is later.	1. IP safety has been evaluated in renal transplant recipients in a Phase 1 study but additional safety data for this IP were requested by the FDA.
**SECONDARY**		
2. Efficacy of the IP for MDRO decolonization	3. MDRO decolonization efficacy will be measured as:<br>i. MDRO cell densities estimated from quantitative stool cultures at Days 7, 14, 21 and 28<br>ii. Proportion of patients positive for any target MDRO cultures at Day 28.	1. MDRO colonization frequently precedes infection and is an important factor in transmission to other susceptible individuals. Based on data from prior studies, we expect a 60% absolute reduction of culture-detected colonization in IP-treated participants.
**EXPLORATORY**		
• Estimate the potential effect size of MT in reducing facility MDRO prevalence.	i. Cumulative MDRO prevalence will be measured as the proportion of sampled patients with one or more positive culture result with a target MDRO (e.g. CRE, ESCRE, VRE, CRPa, *C. difficile*)<br>ii. MDRO category prevalence will be measured as the proportion of sampled patients with a positive culture result for each category.	MDRO-colonized patients are an important source of healthcare transmission. Some patients contribute disproportionately to transmission but the secondary effects of reducing patient MDRO colonization with MT have not been studied.
• Evaluate feasibility of participant recruitment in	Estimate proportion of eligible participants who consent to FMT	This population has a high degree of medical complexity, relies on

OBJECTIVES	ENDPOINTS	ENDPOINT JUSTIFICATION
LTACHs and/or vSNFs.	and culture swab collection (Sentinel REACT), to swabs alone (Group A) or to medical record review alone (Group B).	decision making from legally-authorized representatives (LARs) and has not been previously studied.
• Estimate the potential effect size of MT in reducing facility *C. difficile* infection (CDI) incidence rates.	Compare incidence rates from NHSN reporting and clinical culture results in the two months prior vs the two months post MT.	Treatment of all culture-positive participants with *C. difficile* could measurably reduce facility incidence of *C. difficile*.
• Estimate the potential effect size of MT in reducing facility new MDRO colonization incidence rates at 30 days.	Participants sampled in both pre and post prevalence sampling time points will be compared to explore frequency of new (i.e. negative pre-MT, positive post-MT) isolation of target MDROs.	Treatment of all culture-positive participants with *C. difficile* could measurably reduce facility incidence of target MDROs.
• Estimate the potential effect size of MT in reducing facility MDRO blood stream infection (BSI) incidence rates.	Compare incidence rates from NHSN reporting and clinical culture results in the two months prior vs the two months post MT.	Data from retrospective studies have suggested that FMT may reduce the incidence of blood stream infections, which have a relatively high frequency in LTACHs/vSNFs due to the medical complexity of this population.
• Identify AR genes with the greatest log-fold increase and decrease in patients treated with MT.	Metagenomic analysis of coverage of AR genes in participant samples subjected to shotgun metagenomic sequencing.	Prior work suggests that some AR genes may increase post FMT but most AR genes associated with clinical resistance or epidemiologic concern appear to decrease. Additional study is needed to better understand these dynamics.
• Identify presumptive donor-derived strains that are most frequently detected in MT recipients prior to MDRO decolonization.	Metagenomic analysis of donor-derived strains tracked in recipient metagenomes with strain-tracking tools (e.g. inStrain).	Taxa are thought to be causally associated with decolonization or pathogen reduction but need further study.
• Identify presumptive donor-derived genes that	Metagenomic analysis of donor-derived genes tracked in recipient metagenomes with strain-tracking	Gene-associated functions may be more conserved than taxonomic

OBJECTIVES	ENDPOINTS	ENDPOINT JUSTIFICATION
are most frequently detected in MT recipients prior to MDRO decolonization.	and coverage analysis tools (e.g. inStrain).	composition and causally associated with decolonization or pathogen reduction but need further study.
• Identify differences in MDRO decolonization efficacy by MT donor.	Metagenomic analysis of dissimilarity and differential abundance of taxa and genes that between doses from different donors.	Donors have substantial variability over time in microbiome composition, which may translate to variable dose efficacy, which could clarify causal taxa or functions associated with response.
• Identify differences in MDRO decolonization efficacy by MT manufacturing lot.	Metagenomic analysis of dissimilarity and differential abundance of taxa and genes that between doses from the same donor.	Donors have substantial variability over time in microbiome composition, which may translate to variable dose efficacy, which could clarify causal taxa or functions associated with response.
• Identify potential clusters and pathways of MDRO transmission with genomic, metagenomic, location, and provider badge proximity data.	Comparative genomic analysis of bacterial isolate similarity and clustering with tests of temporospatial overlap of healthcare providers or other healthcare factors that may have allowed transmission.	Prevalence and longitudinal sampling allow precise detection of related isolate genomes that may uncover unrecognized transmission events.

1886

Appendix B: DSMB Charter

Abbreviations

Please refer to the following abbreviations listed below, which are referenced throughout this DSMB Charter:

AE	Adverse Event	IND	Investigational New Drug
COI	Conflict of Interest	MDRO	Multidrug Resistant Organism
Co-I	Co-Investigator	SI	Sponsor Investigator
MT	Microbiota Therapy	DSMB	Data and Safety Monitoring Board
IRB	Institutional Review Board	SAE	Serious Adverse Event
HIPAA	Health Insurance Portability and Accountability Act	Sub-I	Sub-Investigator

DSMB Purpose

This committee's charge is to provide ongoing monitoring for the Sentinel REACT and REACT clinical trial. The committee's major purpose is to ensure the safety of study participants as well as evaluate the ongoing status of the trial by overseeing all aspects of data auditing including validity and integrity.

DSMB Membership

All DSMB members are required to sign a Confidentiality and COI Statement related to the trial discussed. Members will recuse themselves from the discussion and voting if a conflict exists for the given protocol. Quorum requirements: four members, including three voting clinical specialists and one non-voting member. The SI will appoint clinical specialists to the DSMB who have no direct relationship to the trial:

Non-Voting Members:	☐ 1 – Administrator
Voting Members:	☑ 2 – Infectious Diseases Physicians ☐ 1 – Nurse
Key: ☑: Attendance is required to satisfy the quorum requirement.	
☐: Attendance is not required.	

Logistics

The DSMB will meet every six months on average and/or more regularly if required to review toxicity, data submission compliance, participant accrual, protocol compliance, and data quality. An unblinded volunteer will serve as administrative support to the committee. Minutes with action items and recommendations made to the study team will be kept and filed in the regulatory binder and reported to the IRB as necessary. All trial and participant information will remain confidential. The following information will be provided to the committee:

Accrual Statistics	e.g. (Pre-)Screened, screen fails, and trial participants
Adverse Events	e.g., Reported ≥ Grade 2 unexpected AEs and all SAEs
Summary of Deaths	e.g., Occurring 30 days from last intervention, during treatment, and follow-up
Outcome Data	e.g., Outcome measures comparing baseline to post-intervention
Protocol Compliance	e.g., Regulatory compliance and timeliness of data entry
Audit Results	e.g., Audit results from the FDA and/or internal audits of data quality
Other Information	e.g., Laboratory values and participant charts will be provided upon DSMB request

Monitoring Procedures

The SI will ensure that informed consent is obtained prior to performing any research procedures, that all subjects meet eligibility criteria, and that the study is conducted according to the Emory IRB-approved research plan. The SI ensures all protocol deviations and AEs are reported to the Emory IRB according to the applicable regulatory requirements. An independent monitor will review consent forms, study eligibility, safety and efficacy data for the study after the first participant, at six months after the first participant, every 12 months thereafter, and at end of study. Study data are accessible at all times to the study team (SI, Sub-Is, study coordinator) and will be reviewed by the study team as follows:

Real-time	Individual adverse events will be reviewed in real-time.
Weekly Basis	An aggregate of adverse events will be reviewed each week.
Monthly Basis	All study drop-outs and protocol deviations/violations will be reviewed each month.

Data Management Plan

The topics that the DSMB will review as part of the Data Management Plan include, but are not limited to:

Protocol Compliance	Compliance of regulatory documents, study data accuracy, and completeness will be maintained through an internal study team quality assurance process.
Confidentiality	Confidentiality throughout the trial will be maintained in compliance with HIPAA requirements.
Dataset	A dataset will be created in a secure file, on a HIPAA compliant server, and all information will be kept in a locked office, with access given only to study staff.
Participant Tracking List	The list of study participants will contain subject name, birth date, and the number of the corresponding data abstraction sheet. Once the data from all included subjects is entered into a database, the list of identifiable patient information will be destroyed.
Samples	The samples remaining after processing will be kept for future research on those participants who consented to the optional control groups.

Clinical Trial Information

This DSMB Charter is for the following clinical trial:

Protocol ID: **Sentinel - REACT**	Phase: 2	Emory IRB: 00005467	ClinicalTrials.gov NCT: TBD	Protocol: 17Feb2023
IND Product: Allogeneic Microbiota in Glycerol (10%) (AMG)			IND Product Delivery: Feeding tube or rectal instillation	IND Date: 04Jan2023

Protocol Title: Sentinel Cohort for the Response to Emerging Antimicrobial resistance with Containment microbiota restoration Therapy Trial (Sentinel REACT)

Brief Study Overview: Multi-drug resistant organism (MDRO) colonization frequently precedes potentially fatal infections. In addition, colonization promotes transmission to other patients. MDRO colonization thus increases direct healthcare costs of MDRO-related health impacts, and increases indirect costs of CMS penalties in high-prevalence healthcare facilities. Unfortunately, there are no approved therapies for intestinal decolonization. Microbiome therapies such as fecal microbiota transplantation are up to 90% efficacious for reducing culture-detected colonization with multi-drug resistant organisms (MDROs) but they have not yet been evaluated in long-term care facilities. REACT is a CDC-funded clinical trial of microbiome therapy for MDRO-colonized patients in long-term care facilities.

Study Team:

Oversight responsibility is held by the SI/IND-Holder with delegated support by the study team:

SI	Michael H. Woodworth, MD, MSc	mwoodwo@emory.edu	404-234-8452	PIC 37631
Sub-I	Colleen S. Kraft, MD, MSc	colleen.kraft@emory.edu	404-712-8889	PIC 16425
Sub-I	Ahmed Babiker, MBBS	ababike@emory.edu	202-257-3744	

____________________ 17 February 2023

Sponsor-Investigator/IND Holder Signature — Date of Signature

Appendix C: Instructions for Patients Collecting Home Stool/Peri-Rectal Swab Samples

Please collect your samples and call the courier **Monday through Friday before 1pm**.

Call Nurse Amanda at #404-610-6305 if you have any questions/concerns.

Place the gel pack in the freezer, right away, so it is completely frozen when you need it.

**Stool** & **Rectal Swab** Sample Collection Instructions
** ***Wash hands in warm soapy water before you begin*** **
1. Bring your collection kit and supplies into the bathroom with you so that they are easily accessible while you are sitting on the toilet.
1. Empty your bladder into the toilet (not the stool container) before collecting your stool sample. **It is important to avoid mixing the stool sample with urine**.
2. Place Commode Specimen Collection System under toilet seat in center of rear of toilet bowl with lid off.
3. Close toilet seat to hold collection system in place.
4. Sit down on the toilet seat.
5. Remove the e-swab from its packaging, along with the transport tube/container.
6. Insert the “cotton” tip of the e-swab 3 cm into your anus (about the length of your fingertip).
7. Gently swirl the swab around and then remove it.
8. Remove the cap of the transport tube and place the cotton tip end of the swab into the liquid in the tube.
9. Bend the stick to snap it so that it fits into the tube and securely tighten the screw cap.
10. Place the tube into the small biohazard bag that is included in your kit.
11. Defecate into collection system. *Do not place toilet tissue in with the stool sample.*
12. Snap lid onto container - **tightly** - after collection and remove toilet bowl bracket by pushing down on sides. Discard the bracket.

13. **Write the date and time** of stool collection on the label on the container & on the Ziploc bag.
14. Place the container of stool into the Ziploc bag.
15. Place the **sealed** Ziploc bag and the biohazard bag containing the e-swab into the cooler bag with the frozen/cold pack(s).
16. Wash your hands in warm soapy water.
17. Place the cooler bag inside the blue poly-bag.
18. If you are able, store the entire blue poly-bag in the refrigerator (**NOT the freezer**) until pick-up.
19. Call/Text Research Nurse Amanda (# 404-610-6305) and let her know your sample is ready.
20. Please call Courier Express at #770-955-3030 – Mon through Fri **<u>before 1pm</u>**<br>• tell them you have a package ready for a **STAT** pick-up<br>• give them **account number 3124**, they should have the correct delivery location on file.<br>• If not, please request that your sample be delivered to:<br>**101 Woodruff Circle, Suite 7007, Atlanta, GA, 30322**
