# Supplementary material for: Microbiota Transplantation Among Patients Receiving Long-Term Care: The Sentinel REACT Nonrandomized Clinical Trial
Source: JAMA Netw Open. 2025 Jul 24;8(7):e2522740. doi: 10.1001/jamanetworkopen.2025.22740 (PMC12290730; doi:10.1001/jamanetworkopen.2025.22740)
Supplement: Supplement 2. — eMethods. eTable 1. Serious adverse events stratified at participant and event level by body system and relatedness to fecal microbiota transplant product or procedures. eTable 2. Solicited adverse events among fecal microbiota transplant (FMT) recipients eTable 3. Crude difference in antibiotic days of therapy rate per 1000 patient-days between fecal microbiota transplant recipients and untreated multidrug-resistant organism–positive contemporaneous controls over the 6 months before and after prevalence sampling eTable 4. Difference in antibiotic days of therapy rate per 1000 patient-days between fecal microbiota transplant recipients and untreated contemporaneous controls over the 6 months before and after prevalence sampling using a difference-in-differences model eFigure 1. Overall multidrug-resistant organism prevalence among patients admitted to a long-term acute care hospital in 2023 in 2 prevalence surveys 35 days apart to screen for eligibility for the Sentinel REACT trial eFigure 2. Summary of respiratory culture isolate count and identification in the 6 months before and after prevalence sampling stratified by fecal microbiota transplantation receipt vs untreated contemporaneous controls eFigure 3. Summary of urine culture isolate count and identification in the 6 months before and after prevalence sampling stratified by fecal microbiota transplantation receipt vs untreated contemporaneous controls eFigure 4. Summary of positive clinical microbiology cultures per participant among fecal microbiota transplantation recipients vs untreated multidrug-resistant organism–positive contemporaneous controls in 6 months before compared to 6 months after prevalence survey sampling by participant and specimen type eReferences. [file jamanetwopen-e2522740-s002.pdf]

Supplemental Online Content

Woodworth MH, Babiker A, Prakash-Asrani R, et al. Microbiota transplantation among patients receiving long-term care: the Sentinel REACT nonrandomized clinical trial. *JAMA Netw Open*. 2025;8(7):e2522740. doi:10.1001/jamanetworkopen.2025.22740

eMethods.
eTable 1. Serious adverse events stratified at participant and event level by body system and relatedness to fecal microbiota transplant product or procedures.
eTable 2. Solicited adverse events among fecal microbiota transplant (FMT) recipients
eTable 3. Crude difference in antibiotic days of therapy rate per 1000 patient-days between fecal microbiota transplant recipients and untreated multidrug-resistant organism–positive contemporaneous controls over the 6 months before and after prevalence sampling
eTable 4. Difference in antibiotic days of therapy rate per 1000 patient-days between fecal microbiota transplant recipients and untreated contemporaneous controls over the 6 months before and after prevalence sampling using a difference-in-differences model
eFigure 1. Overall multidrug-resistant organism prevalence among patients admitted to a long-term acute care hospital in 2023 in 2 prevalence surveys 35 days apart to screen for eligibility for the Sentinel REACT trial
eFigure 2. Summary of positive clinical microbiology cultures per participant among fecal microbiota transplantation recipients vs untreated multidrug-resistant organism–positive contemporaneous controls in 6 months before compared to 6 months after prevalence survey sampling by participant and specimen type
eFigure 3. Summary of respiratory culture isolate count and identification in the 6 months before and after prevalence sampling stratified by fecal microbiota transplantation receipt vs untreated contemporaneous controls
eFigure 4. Summary of urine culture isolate count and identification in the 6 months before and after prevalence sampling stratified by fecal microbiota transplantation receipt vs untreated contemporaneous controls
eReferences.

This supplemental material has been provided by the authors to give readers additional information about their work.

© 2025 Woodworth MH et al. *JAMA Network Open*.

eMethods

Intervention

FMT doses were manufactured with stool from screened healthy donors at Emory under banking protocols reviewed by FDA and approved by the Emory IRB and previously described.[1] FMT was instilled as a 250mL suspension (average 10^{11} anaerobic CFU / dose) via percutaneous gastrostomy tube (as divided 60mL syringe bolus instillations) or retention enema. For feasibility and ease of administration in this patient population with a high proportion receiving nutrition via gastrostomy feeding tube, the protocol allowed for either route. When a participant vomited most of the administered dose instilled via gastrostomy tube, treatment via enema in the second group was encouraged. After treatment, participants were observed for 30 minutes for immediate adverse events (AEs). Because this population frequently receives broad-spectrum antibiotic therapy, no enteric microbiota conditioning medications (e.g., antibiotics, laxatives, or anti-motility agents) were administered prior to instillation via either route.

Microbiology Methods

ESwab (COPAN Diagnostics, Murrieta, CA, USA) eluates were plated on differential and selective culture media for qualitative MDRO detection.[2] Participant microbiologic outcomes were assessed by peri-rectal ESwab cultures at baseline (APPS prevalence sampling) and weekly from Day 0 (day of FMT collected prior to treatment) to Day 28. Suspected *C. difficile* isolates underwent PCR assays with primers for detection of the pan-*C. difficile rplP* gene and *tcdB* toxin gene targets as previously described to further classify isolates as toxigenic or non-toxigenic.[3,4] For non-*C. difficile* positive cultures, pure-growth colonies were identified by MALDI-TOF mass spectrometry (bioMérieux, Marcy-l'Étoile, France) with susceptibility testing performed by Vitek2 microdilution (bioMérieux, Marcy-l'Étoile, France) or Oxoid™ 30µg vancomycin disc diffusion (Thermo Fisher Scientific) (to classify *Enterococcus spp* as VRE or vancomycin-susceptible *Enterococcus spp*) as per CLSI.[5]

Table e1: *C. difficile* Polymerase Chain Reaction (PCR) Primers

Target/Citation	Direction	Sequence (5’ -> 3’)
rplP[3]	Forward	ACCAGCAGAGACTCGTATGG
	Reverse	GTGCAGCAAGTCTCATAGCTTC
tcdB[4]	Forward	GTGTAGCAATGAAAGTCCAAGTTTACGC
	Reverse	CACTTAGCTCTTTGATTGCTGCACCT

Metagenomic Methods

According to manufacturer instructions, peri-rectal swab DNA was extracted with ZymoBIOMICS MagBead DNA kits, libraries were prepared with Nextera XT kits, and after quality control, pooled DNA libraries were sequenced on an Illumina NovaSeq 6000 instrument by the Emory Non-Human Primate Research Center Genomics Core Facility. Negative template (molecular water) controls were included with each extraction batch and sequenced. Raw reads were processed with Trimmomatic and potential human reads that aligned to the human reference genome hg38 were removed with bowtie2.[6,7] Remaining read taxonomic classification was performed with kraken2/bracken and analyzed after removing taxa that were detected at low abundance in negative control libraries with the phyloseq package in R (R Foundation for Statistical Computing, Vienna, Austria) using the R Studio interface (Boston, MA).[8–10] Species alpha diversity was assessed by the inverse Simpson index, which accounts for the number of species and their evenness in abundance. Participants were classified with intestinal domination if ≥30% of species-level classified shotgun metagenomic reads aligned to a single bacterial pathogen that had been cultured from the cohort, adapted from the definition of Taur et al. for 16S rRNA gene amplicon analysis.[11] These species included: *Citrobacter braakii*, *Clostridioides difficile, Enterobacter cloacae* complex, *Enterococcus spp, Escherichia coli, Klebsiella oxytoca, Klebsiella pneumoniae, Proteus mirabilis, Pseudomonas aeruginosa, Pseudomonas flurescens, Stenotrophomonas maltophilia.*

Difference in Differences Statistical Methods

The impact of FMT over time was also evaluated using a Difference-in-Differences approach.[12] This method incorporates baseline differences to estimate the effect size of an intervention while also accounting for effects other than the intervention. The Difference in Differences of antibiotic days of therapy after FMT was modeled as a function of time, FMT treatment, and their interaction. A participant-specific fixed effect was used to control for unobserved, time-invariant characteristics. Robust standard errors were used to address heteroskedasticity, and standard errors were clustered at the participant level to account for repeated measurements within the same

participant. The coefficient estimates, 95% confidence intervals, and p-values were reported. Results were described or analyzed for statistical significance in R using the R Studio Interface with alpha level of 0.05.

© 2025 Woodworth MH et al. *JAMA Network Open*.

eTable 1: Serious adverse events stratified at participant and event level by body system and relatedness to fecal microbiota transplant (FMT) product or procedures.

	Participants (N=10)	
**Body System**	**Related[A]**	**Unrelated[B]**
Respiratory	0	5
Cardiovascular	0	2
Gastrointestinal	0	1
Metabolic and Nutritional	0	1

	Serious Adverse Events (N=14)	
**Body System**	**Related[A]**	**Unrelated[B]**
Respiratory	0	10
Cardiovascular	0	2
Gastrointestinal	0	1
Metabolic and Nutritional	0	1

A – sum of adverse events classified as probably or definitely related to IP or instillation procedure. B – sum of adverse events classified as not related, unlikely to be related, or potentially related (when only relationship was occurrence after treatment).

© 2025 Woodworth MH et al. *JAMA Network Open*.

eTable 2: Solicited Adverse Events (AEs) among fecal microbiota transplant (FMT) recipients.

Solicited AE	D0 Baseline	D0 Post FMT	D1	D2	D3	D4	D5	D6	D7
**Fever**	1 (9.1%)	0 (0%)	0 (0%)	0 (0%)	0 (0%)	0 (0%)	0 (0%)	0 (0%)	0 (0%)
**Vomiting**	0 (0%)	1 (9.1%)	0 (0%)	0 (0%)	0 (0%)	0 (0%)	0 (0%)	0 (0%)	0 (0%)
**Abdominal Pain**	0 (0%)	0 (0%)	0 (0%)	0 (0%)	1 (9.1%)	0 (0%)	0 (0%)	0 (0%)	1 (9.1%)
**Bloating**	0 (0%)	0 (0%)	0 (0%)	0 (0%)	0 (0%)	0 (0%)	0 (0%)	0 (0%)	1 (9.1%)
**Constipation**	0 (0%)	0 (0%)	0 (0%)	0 (0%)	0 (0%)	0 (0%)	0 (0%)	0 (0%)	1 (9.1%)
**Diarrhea**	0 (0%)	1 (9.1%)	0 (0%)	0 (0%)	0 (0%)	0 (0%)	0 (0%)	0 (0%)	1 (9.1%)
**Flatulence**	1 (9.1%)	0 (0%)	0 (0%)	0 (0%)	0 (0%)	0 (0%)	0 (0%)	0 (0%)	1 (9.1%)

Note: one participant was treated twice (with >30 days between treatments). Data reflect 11 treatments of 10 unique participants.

© 2025 Woodworth MH et al. *JAMA Network Open*.

eTable 3: Crude difference in Antibiotic Days of Therapy rate per 1,000 patient days between fecal microbiota transplant (FMT) recipients and untreated multidrug-resistant organism (MDRO) positive contemporaneous controls over the six months before and after prevalence sampling. (n = 42)

	**FMT (N=10)**	**MDRO-positive controls (N=32)**
Six months before sampling	45.5	30.5
Six months after sampling	18.9	29.7
Difference	-26.6	-0.8
**Difference in Differences**	-26	

© 2025 Woodworth MH et al. *JAMA Network Open*.

eTable 4: Difference in antibiotic days of therapy rate per 1,000 patient days between fecal microbiota transplant (FMT, N=10) recipients and untreated contemporaneous controls (N=32) over the six months before and after prevalence sampling using a difference-in-differences model. (n = 42)

**Parameter**	Estimate	95% CI	P-value
Time * FMT	-26	-64 to 12	0.18

© 2025 Woodworth MH et al. *JAMA Network Open*.

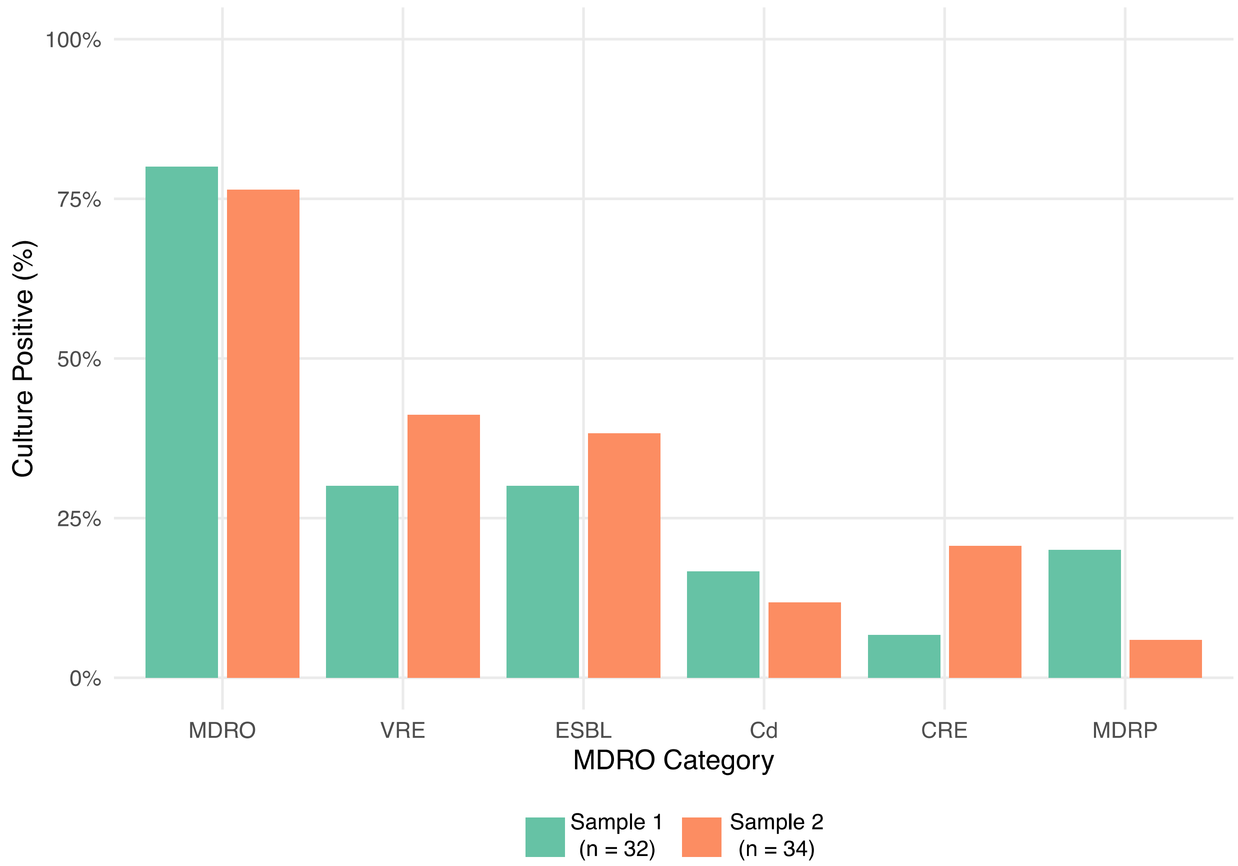

eFigure 1 Overall multidrug-resistant organism (MDRO) prevalence is high among patients admitted to a long-term acute care hospital in 2023 in two prevalence surveys 35 days apart to screen for eligibility for the Sentinel REACT trial. MDRO column indicates participant-level composite detection of all MDRO categories. ESBL – extended-spectrum beta-lactamase producing *Enterobacterales,* VRE – vancomycin-resistant *Enterococcus*, MDRP – multidrug-resistant *Pseudomonas*, CD – *C. difficile*.

© 2025 Woodworth MH et al. *JAMA Network Open*.

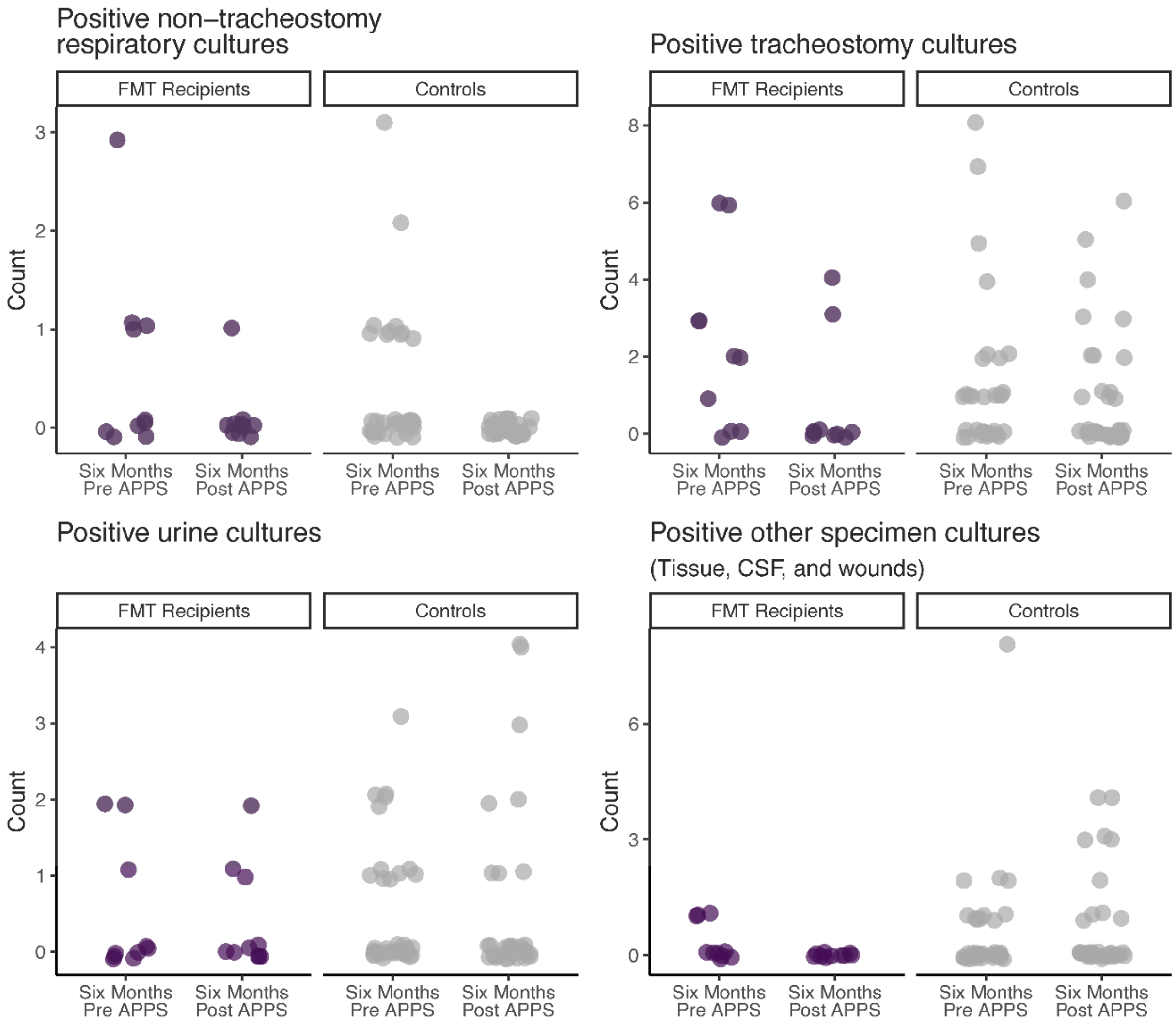

eFigure 2 Summary of positive clinical microbiology cultures per participant among fecal microbiota transplantation (FMT) recipients (N=10) vs untreated multidrug-resistant organism (MDRO) positive contemporaneous controls (N=32) in six months pre compared to six months post prevalence survey sampling by participant, and specimen type.

© 2025 Woodworth MH et al. *JAMA Network Open*.

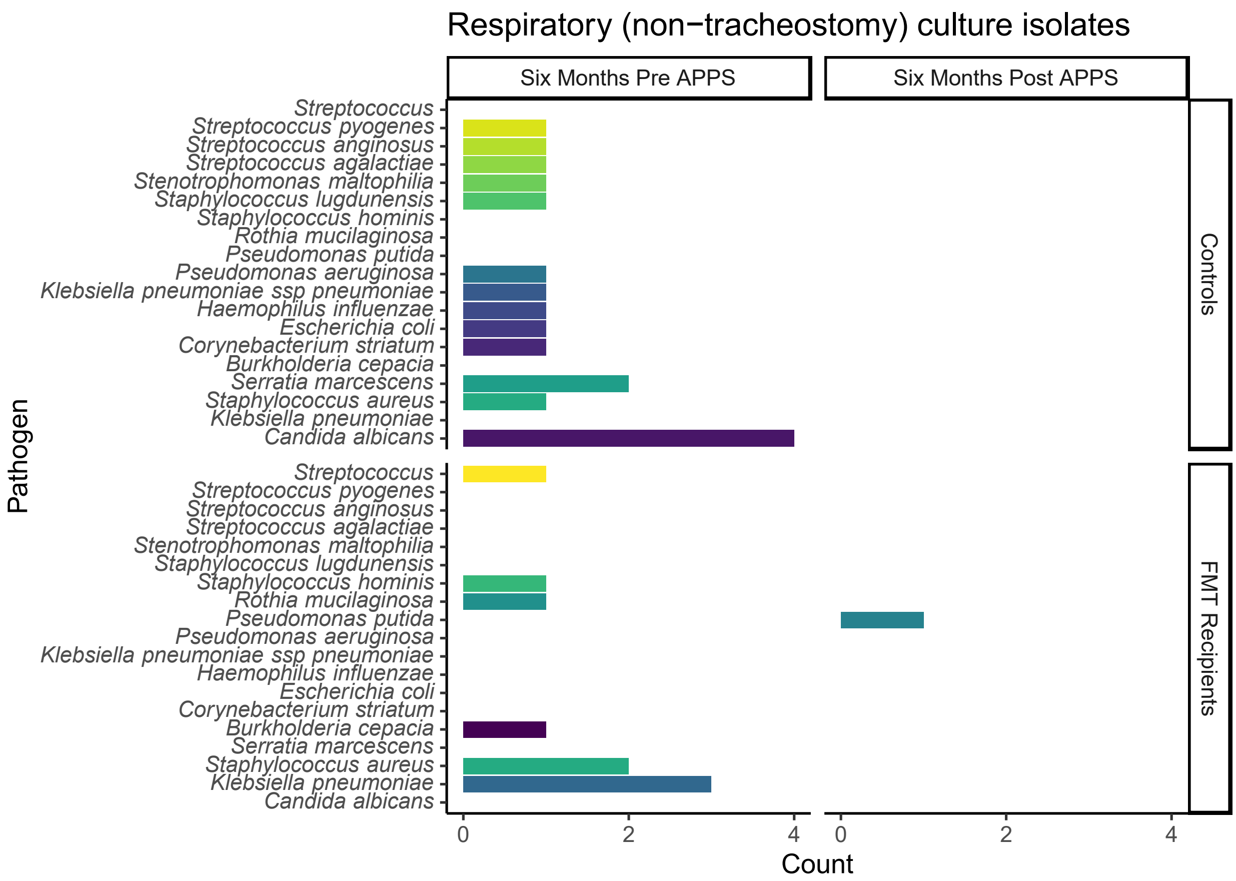

eFigure 3 Summary of respiratory culture isolate count and identification in the six months before and after prevalence sampling stratified by fecal microbiota transplantation (FMT) receipt vs untreated contemporaneous controls. Colors represent pathogen species.

© 2025 Woodworth MH et al. *JAMA Network Open*.

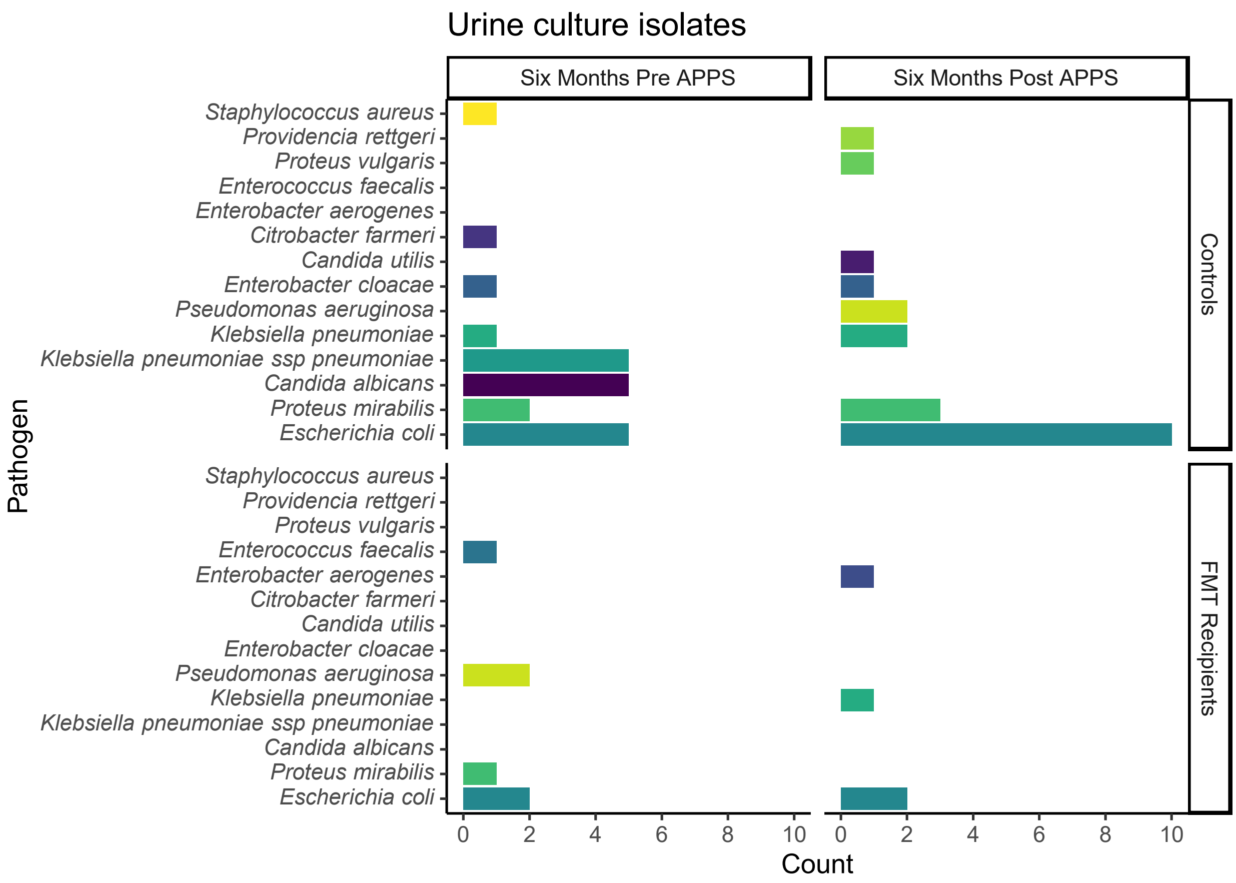

eFigure 4 Summary of urine culture isolate count and identification in the six months before and after prevalence sampling stratified by fecal microbiota transplantation (FMT) receipt vs untreated contemporaneous controls. Colors represent pathogen species.

© 2025 Woodworth MH et al. *JAMA Network Open*.

eReferences

1. Woodworth MH, Conrad RE, Haldopoulos M, et al. Fecal microbiota transplantation promotes reduction of antimicrobial resistance by strain replacement. *Sci Transl Med*. 2023;15(720). doi:10.1126/scitranslmed.abo2750
2. Magiorakos AP, Srinivasan A, Carey RB, et al. Multidrug-resistant, extensively drug-resistant and pandrug-resistant bacteria: an international expert proposal for interim standard definitions for acquired resistance. *Clin Microbiol Infect*. 2012;18(3):268-281. doi:10.1111/j.1469-0691.2011.03570.x
3. VanInsberghe D, Elsherbini JA, Varian B, Poutahidis T, Erdman S, Polz MF. Diarrhoeal events can trigger long-term Clostridium difficile colonization with recurrent blooms. *Nat Microbiol*. 2020;5(4):642-650. doi:10.1038/s41564-020-0668-2
4. Larson AM, Fung AM, Fang FC. Evaluation of *tcdB* Real-Time PCR in a Three-Step Diagnostic Algorithm for Detection of Toxigenic *Clostridium difficile*. *J Clin Microbiol*. 2010;48(1):124-130. doi:10.1128/JCM.00734-09
5. CLSI (Clinical and Laboratory Standards Institute). *Performance Standards for Antimicrobial Susceptibility Testing*.; 2024.
6. Bolger AM, Lohse M, Usadel B. Trimmomatic: A flexible trimmer for Illumina sequence data. *Bioinformatics*. 2014;30(15):2114-2120. doi:10.1093/bioinformatics/btu170
7. Langmead B, Salzberg SL. Fast gapped-read alignment with Bowtie 2. *Nat Methods*. 2012;9(4):357-359. doi:10.1038/nmeth.1923
8. Lu J, Rincon N, Wood DE, et al. Metagenome analysis using the Kraken software suite. doi:10.1038/s41596-022-00738-y
9. McMurdie PJ, Holmes S. phyloseq: An R Package for Reproducible Interactive Analysis and Graphics of Microbiome Census Data. Watson M, ed. *PLoS One*. 2013;8(4):e61217. doi:10.1371/journal.pone.0061217
10. Oksanen J, Blanchet FG, Friendly M, et al. vegan: Community Ecology Package. Published online 2020. https://cran.r-project.org/package=vegan
11. Taur Y, Xavier JB, Lipuma L, et al. Intestinal domination and the risk of bacteremia in patients undergoing allogeneic hematopoietic stem cell transplantation. *Clin Infect Dis*. 2012;55(7):905-914. doi:10.1093/cid/cis580
12. Streeter AJ, Lin NX, Crathorne L, et al. Adjusting for unmeasured confounding in nonrandomized longitudinal studies: a methodological review. *J Clin Epidemiol*. 2017;87:23-34. doi:10.1016/j.jclinepi.2017.04.022

© 2025 Woodworth MH et al. *JAMA Network Open*.
