# Supplementary material for: Microbiota Transplantation Among Patients Receiving Long-Term Care: The Sentinel REACT Nonrandomized Clinical Trial
Source: JAMA Netw Open. 2025 Jul 24;8(7):e2522740. doi: 10.1001/jamanetworkopen.2025.22740 (PMC12290730; doi:10.1001/jamanetworkopen.2025.22740)
Supplement: Supplement 3. — Data Sharing Statement [file jamanetwopen-e2522740-s003.pdf]

Data Sharing Statement

Woodworth. Microbiota Transplantation Among Patients Receiving Long-Term Care. *JAMA Netw Open*. Published July 24, 2025. doi:10.1001/jamanetworkopen.2025.22740

Data
Additional Information: clinicaltrials.gov, NCT05780801
Data available: Yes
Data types: Deidentified participant data, Data dictionary
How to access data: Deidentified participant data and data dictionary were uploaded to a zenodo repository available at 10.5281/zenodo.15710925. Metagenomic data were uploaded to dbGaP under accession ID phs004084.v1.p1.
When available: With publication

Supporting Documents
Document types: None

Additional Information
Who can access the data: Metagenomic data at dbGaP will require data access request through their procedures. Deidentified participant data and data dictionary will be unrestricted.
Types of analyses: For any purpose.
Mechanisms of data availability: Metagenomic data at dbGaP will require data access request through dbGaP procedures.
